# Persistent Biases in Binocular Rivalry Dynamics within the Visual Field

**DOI:** 10.3390/vision1030018

**Published:** 2017-06-29

**Authors:** Kevin C. Dieter, Jocelyn L. Sy, Randolph Blake

**Affiliations:** Vanderbilt Vision Research Center and Department of Psychology, Vanderbilt University, Nashville, TN 37240, USA

**Keywords:** binocular rivalry, bistable vision, individual differences, 3D vision

## Abstract

Binocular rivalry is an important tool for measuring sensory eye dominance—the relative strength of sensory processing in an individual’s left and right eye. By dichoptically presenting images that lack corresponding visual features, one can induce perceptual alternations and measure the relative visibility of each eye’s image. Previous results indicate that observers demonstrate reliable preferences for several image features, and that these biases vary within the visual field. However, evidence about the persistence of these biases is mixed, with some suggesting they affect only the onset (i.e., first second) of rivalry, and others suggesting lasting effects during prolonged viewing. We directly investigated individuals’ rivalry biases for eye and color within the visual field and interestingly found results that mirrored the somewhat contradictory pattern in the literature. Each observer demonstrated idiosyncratic patterns of biases for both color and eye within the visual field, but consistent, prolonged biases only for the eye of presentation (sensory eye dominance, SED). Furthermore, the strength of eye biases predicted one’s performance on a stereoacuity task. This finding supports the idea that binocular rivalry and other binocular visual functions may rely on shared mechanisms, and emphasizes the importance of SED as a measure of binocular vision.

## 1. Introduction

We view the world through two laterally separated eyes, yet we see a single, unified visual world. Simple though this observation may be, it highlights a critical and complex transformation: oculomotor mechanisms and neural processes within early stages of the visual pathway work synergistically to establish matches between optical features imaged separately on the left and right retinae and to combine the neural representations of those features into a single, 3D cyclopean impression of the visual world. Not only do these processes fuse multiple inputs into a unified view of the world, but they also make explicit 3D perspective relations among objects that are not represented in either eye’s image alone. This aspect of binocular vision is strikingly demonstrated by our experience of stereopsis—perception of depth in the visual scene that arises from neural computations relating to the distance (“disparity”) between where given object features are imaged in the left and right eye’s retinal coordinates [1,2]. Importantly, these processes also give rise to a host of other improvements in human visual performance, including enhanced contrast and luminance sensitivity, more accurate visually guided reaching, and even faster reading [3,4,5,6,7]. Taken together, these uniquely binocular qualities of human vision highlight that the neural mechanisms combining visual information from the separate eyes are critical to good visual function.

However, this process of binocular combination can break down when the two eyes receive inputs that are not suitably matched in terms of sensory strength. For example, presenting images that differ in left and right eye contrast impairs stereoacuity (visual sensitivity to binocular disparity, [8,9]), as does blurring the input to one eye relative to the other [10,11,12]. Such impairments can also arise due to intrinsic imbalances between the eyes, perhaps owing to clinical disorders of binocular vision such as amblyopia [13]. Using the paradigm of binocular rivalry, in which very different images are shown to an observer’s eyes to instigate perceptual alternations, one can quantify the degree of sensory eye dominance (SED) by comparing the frequency [14] or total time [15] for which an individual sees the stimulus presented to the left and to the right eye. This approach reveals that intrinsic imbalances between the eyes are also found in observers who report otherwise normal binocular visual function [15,16,17,18]. Moreover, stereoacuity appears to be strongly related to the degree of (im)balance between the two eyes [19], a potentially important finding that could link stereopsis, binocular fusion and binocular rivalry.

The level of balance between the eyes (as assessed by binocular rivalry) has largely been studied using relatively small, foveally viewed rival stimuli. However, a body of evidence points to a critical involvement of early cortical stages of visual processing in resolving binocular rivalry conflict [20,21,22,23,24], suggesting that binocular interactions may operate with a large degree of local variability. In particular, because neurons in early visual areas of cortex respond to a relatively small region of visual space (i.e., those neurons have small receptive fields), the mechanisms underlying binocular combination in one region of the visual field may be distinct from those underlying the same process in a region of the visual field some distance removed. If SED arises from these potentially heterogeneous mechanisms, it raises the possibility that intrinsic biases in SED measured at the fovea may actually vary as one tests within different visual field locations for the same observer. Indeed, there is existing evidence that the level of balance between the two eyes appears to vary idiosyncratically within different regions of an observer’s visual field, with the SED within a particular “zone” of the visual field predicting an observer’s stereoacuity [25].

What remains unclear is whether these local visual field biases persist during prolonged binocular viewing of rival images. In particular, the finding of pronounced variations in SED across small regions of visual space in some individuals [25] was derived primarily using brief stimulus presentations of both rival stimuli (500 ms) and stereoacuity stimuli (200 ms). Within foveal vision, these measurements taken with brief rivalry stimuli were strongly correlated with the dynamics of rivalry when tracked over longer, 30-s viewing periods [25]. At the same time, however, reliable biases within observers’ peripheral visual fields that are seen when colored dichoptic images are briefly presented (i.e., local “zones” of red or green color preference) do not persist over longer tracking periods. This has led to the suggestion that the “onset” phase of binocular rivalry (i.e., the initial 1-s after the presentation of rivalry images) is distinct from ongoing dynamics [26,27]. Consistent with this distinction, very brief presentations of dichoptic stimuli (~150 ms) lead to “abnormal fusion” of rival patterns [28,29], with brief rivalry presentations characterized by stronger influences of visual attention than are seen across prolonged rivalry tracking blocks [30].

We addressed this disconnect between patterns of rivalry biases for color and eye by asking in the present study if observers demonstrated reliable biases in binocular rivalry dynamics that (1) persisted during prolonged viewing of stimuli and (2) were unique to particular regions of an observer’s visual field. Therefore, we tested whether one eye, or one color, systematically and reliably dominated an observer’s visual perception within a particular region of the visual field. To this end, we asked observers to track binocular rivalry dynamics while viewing stimuli imaged at any one of many different locations within the visual field. Similar to the results of [25,27], we found that observers’ tracking records show evidence for idiosyncratic, but highly reliable, zones of eye dominance and color dominance—that is, within a particular region of the visual field, an observer experienced persistent biases. Interestingly, while ”onset” and prolonged biases for eye (SED) were strongly related, this was not the case for color dominance, where initial biases were not related to those that persisted across prolonged viewing of rival stimuli [27]. Furthermore, we also show (as in [25]) that SED biases are related to the quality of stereoacuity within the corresponding region of the visual field. These biases varied across different locations within each observer’s visual field, creating a mosaic that was unique for each individual but reliable over time. Taken together, the pattern within our data set actually mimics the complex pattern seen in the literature, and further suggests differential effects of eye and color on binocular rivalry dynamics over time. In addition, the spatial and temporal characteristics of these biases pose a methodological challenge for experimental designs in which multiple rivalry stimuli are simultaneously imaged on screen across various peripheral locations.

## 2. Results

### 2.1. Experiment 1 Results

**Figure 1:**
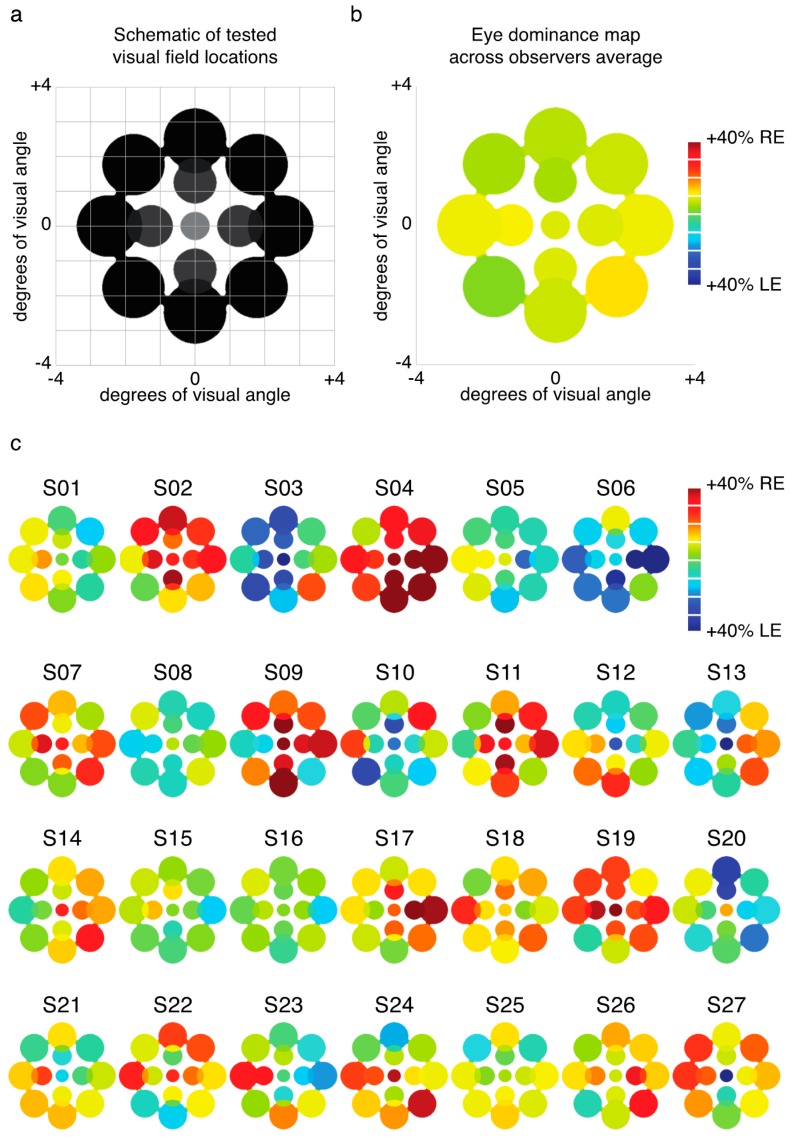
Heat maps of Sensory Eye Dominance (SED): (**a**) Schematic illustrating the 13 visual field locations at which rivalry dynamics were tested for each observer. During each tracking block, orthogonal rotating gratings were presented to the two eyes at one of these 13 positions while observers maintained fixation on a fusion image at the center of the screen; (**b**) Averaging across all individuals for each location yielded no consistent pattern of SED at any tested location. All heat maps are colored so that warm colors indicate right eye (RE) dominance, and cool colors indicate left eye (LE) dominance; (**c**) However, individuals occassionally experienced large changes in SED even for nearby test locations across trials. These patterns appear to be unique, but are consistent within observers across repeated testing sessions.

We examined the relative predominance of each observer’s left eye (LE) and right eye (RE; SED) while that observer viewed binocular rivalry within a circular aperture at one of thirteen locations within the visual field (Figure 1a). Visual inspection of a heat map of SED produced by averaging data for each tested location across all observers suggests relatively uniform SED across visual field locations (Figure 1b). However, looking at the individual data (Figure 1c), it is clear that this result is an artifact of averaging across highly idiosyncratic patterns of SED within individuals’ visual fields. For many observers, local SED varied dramatically even within relatively nearby neighborhoods in visual space (for example see S10 in Figure 1c).

**Figure 2:**
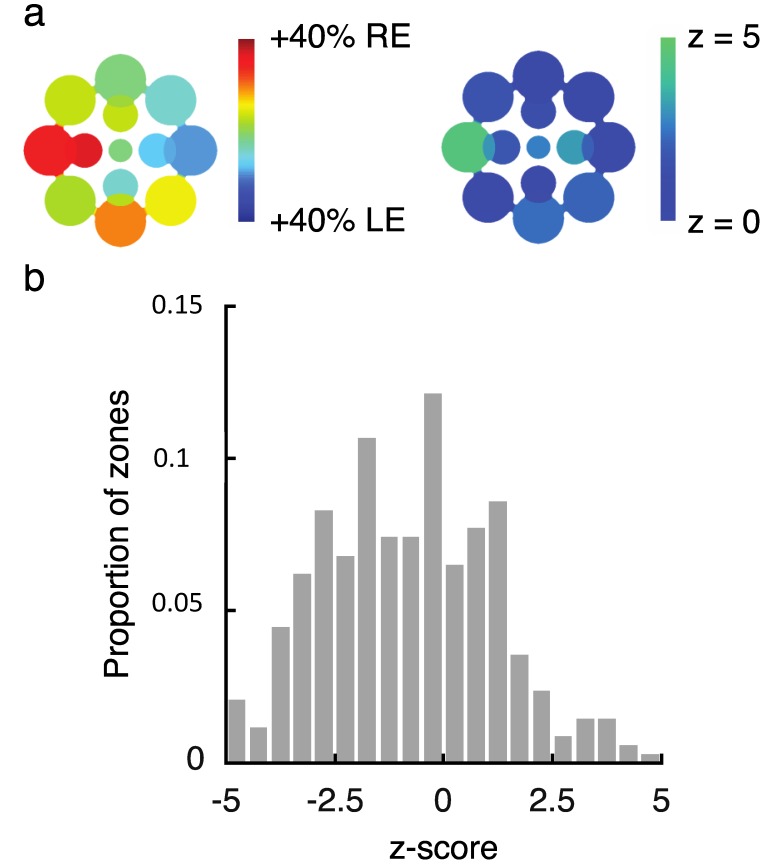
Statistical reliability of SED maps: (**a**) We conducted a bootstrap analysis to estimate the likelihood of observing an SED value in a particular zone that was at least at extreme as the value we obtained, based on the pool of all LE and RE durations for that observer (see Methods). For this sample observer (S23), we found that 1 tested location (2.5 degrees eccentricity, directly left of fixation) had an SED that was very unlikely (absolute value of z > 4) to have arisen from chance. (**b**) The overall distribution of z-scores across all tested locations and observers indicated that 22.8% of zones had a z-score more extreme than ±2.9.

While this variability is notable, we need to confirm that it reflects true differences between visual field locations for these observers and is not simply due to noise across multiple repeated measurements. Using a resampling procedure (see Section 4.1.5), we tested the likelihood of drawing samples as extreme as the observed SED values for each zone (see Figure 2a for results from an illustrative observer). Here, we created separate pools of all LE and RE data for that observer across all zones and then drew 10,000 random (with replacement) samples matched to the number of LE and RE data points in each zone to obtain a z-score for each zone. Because we repeated this analysis 13 times for each observer (once for each zone), we used the Bonferoni-corrected p-value (0.0038) as a benchmark for statistical significance (which corresponds to a z-score of magnitude 2.9 or greater). This analysis revealed that in total, 80/351 (22.8%) tested locations had z-scores of magnitude 2.9 or greater (Figure 2b shows the full distribution of z-scores), indicating that an observed SED value was highly unlikely to have been produced by chance based on the observer’s overall tendency to report left eye (LE) or right eye (RE) across all tested locations. In total, 20/27 (74.1%) of observers had at least 1 zone with a z-score of magnitude 2.9 or greater, with an average of 3.0 ± 3.0 zones/observer. Notably, this analysis only tells us about whether a particular zone differed in SED from that observer’s overall tendency (averaged across all locations) to report the LE or RE as dominant. On its own, therefore, the analysis does not tell us whether a given zone was balanced or unbalanced in SED (i.e., SED close to or far from zero, respectively).

We next asked whether each observer had a significant eye bias overall—that is, whether exclusive dominance was more likely for one eye’s image than the other’s when considered across all tested locations. To conduct this analysis we employed a similar resampling procedure, now pooling all exclusive durations (across both eyes and all tested locations) and drawing 10,000 random (with replacement) bootstrap samples to estimate SED (see Section 4.1.5). Overall, this pooled SED was weak but slightly biased toward the right eye (across observers mean 7.5 ± 13.2%). This value was significant (at z-score > 3 threshold) for 10/27 (37.0%) observers. Both the overall prevalence of unbalanced SED (37.0%) and the stronger tendency for RE dominance (9/10 significantly unbalanced subjects were RE dominant) are consistent with previous findings for foveal vision [15].

**Figure 3:**
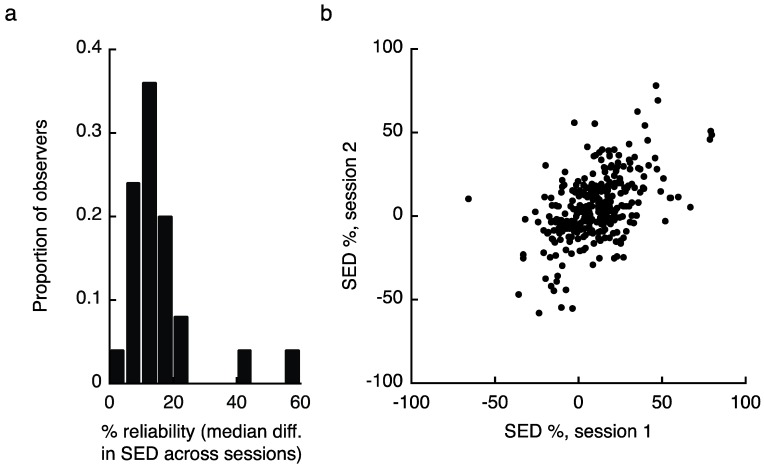
Reliability of SED zones: Two illustrations of the reliability of SED our results across sessions. (1) First, we calculated the absolute value of the difference in observed SED from session 1 to session 2 for each of the 13 tested locations, and took the median value for each observer (N = 25) as their measure of reliability. (**a**) shows the histogram of these values. We found that reliability was generally good, but that two observers were significant outliers (rightmost two bars). (2) We also plotted the SED value obtained in Session 1 against that obtained in Session 2 for each location for each observer. This reveals a strong relationship suggesting strong test-retest reliability of SED maps ((**b**) excludes the two unreliable outliers, N = 23).

Establishing the reality of variable zones of eye dominance in the visual field also requires an analysis of the stability of those putative zones over time. We measured SED zones for each observer in two separate sessions (mean 8.6 ± 5.3 days between tests) and assessed the consistency of the results in three ways (note that S09 and S11 completed only one testing session, and are therefore not included in these analyses). First, we compared the absolute value difference in measured SED value at each of the 13 tested locations for each observer, taking the median value across locations as an estimate of reliability. Overall, SED values in the second testing session were within 15.8 ± 11% of the values obtained during session one (Figure 3a shows the reliability histogram). However, we identified two individuals whose reliability was more than 4 standard deviations worse than the mean of the other 23 observers (two rightmost bars in Figure 3a). Excluding those individuals yielded an across observer reliability of 13.0 ± 4.8%. Note that removing these individuals from the previously reported analyses does not substantially change the results (for reference, these individuals are S06 and S13 in Figure 1c). As a second test of reliability, we produced a scatter plot by taking all measured SED values from session one and plotting them against the same values from session two (Figure 3b; note that this figure excludes the two outliers). While these analyses provide intuitive illustrations of our reliability results, they also assume independence between SED values observed from the same individual. To address this issue we conducted a multilevel linear mixed effects model (e.g., [31]) that assessed the effects of sesson 1 scores on session 2 scores while accounting for non-independence within subjects by specifying a random effect for subjects (SAS code and results available through our figshare repository). This model also disaggregated the effects of session 1 scores to within- and between-subjects components by including a predictor variable in which session 1 scores were centered (i.e., mean-corrected) on a within-subjects basis and another predictor that represented the average of that individual’s scores across the 13 visual field zones. The model was estimated by restricted maximum likelihood (REML). This model indicated that session 1 scores positively predicted session 2 scores, on both a within-subjects (F_1,287_ = 11.98, *p* = 0.0006, B = 0.23) and between-subjects (F_1,287_ = 7.36, *p* = 0.0071, B = 0.56) basis. In addition a second mixed effects model that added interaction terms between the two session 1 predictors and visual field zone indicated no significant interaction effects for either (ps = 0.47 and 0.25, for the within session 1 X zone and between session 1 X zone interactions, respectively). These latter effects indicate that the effects of session 1 on session 2 scores were not conditional upon visual field zone. Finally, we note that our results in Experiments 2 and 3, where we use these Experiment 1 results to make predictions about observer’s performance on different task, offer further confirmation of the consistency of SED zones over time.

**Figure 4:**
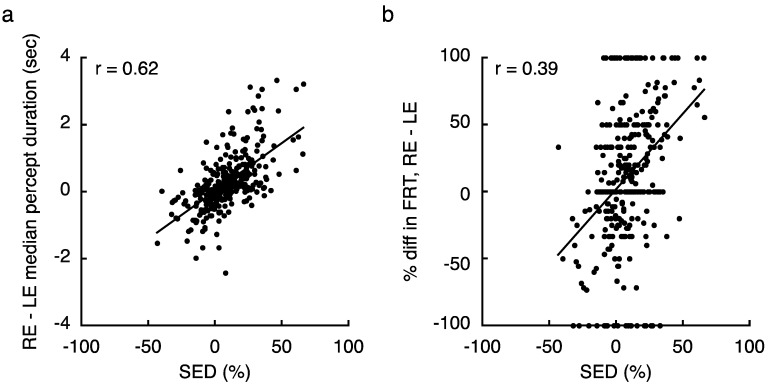
Signatures of SED in binocular rivalry dynamics: As further support for the SED maps reported in Experiment 1, we examined signatures of binocular rivalry dynamics that are associated with SED (see [15]). (**a**) We found that the SED value, obtained by comparing the proportion of predominance of each eye at each location for each observer, was strongly related to the difference in median percept duration between the two eyes. (**b**) The overall SED value at each location also predicted the fraction of return transitions (FRT). Specifically, when one eye was stronger in overall SED, observers tended to experience more return transitions to that eye over the course of extended binocular rivalry viewing.

As one further means of confirming the presence of true regional SED variability, we looked for signature changes in rivalry dynamics that are associated with imbalances between the two eyes in foveal vision [15]. First, we found that SED was strongly correlated with the difference between RE and LE dominance durations within a given visual field zone (r = 0.62, *p* < 10^−34^; Figure 4a). Notably, we also recently demonstrated that large SED values are associated with frequent return transitions to the dominant eye—that is, when one eye is considerably stronger than the other, an observer will often experience dominance of that eye, followed by a period of mixed perception, then followed by a second instance of dominance of the same eye [32]. We indeed found that when tracking rivalry in zones with stronger magnitudes of SED, individuals tended to experience more return transitions overall (Spearman Rank-Order Correlation; r = 0.17, *p* = 0.002). While that metric ignores which eye was returned to, the relationship is even stronger when considering the relative proportion of return transitions to the LE and RE (Spearman Rank-Order Correlation; r = 0.39, *p* < 10^−7^; Figure 4b, see Section 4.1 for metric details).

**Figure 5:**
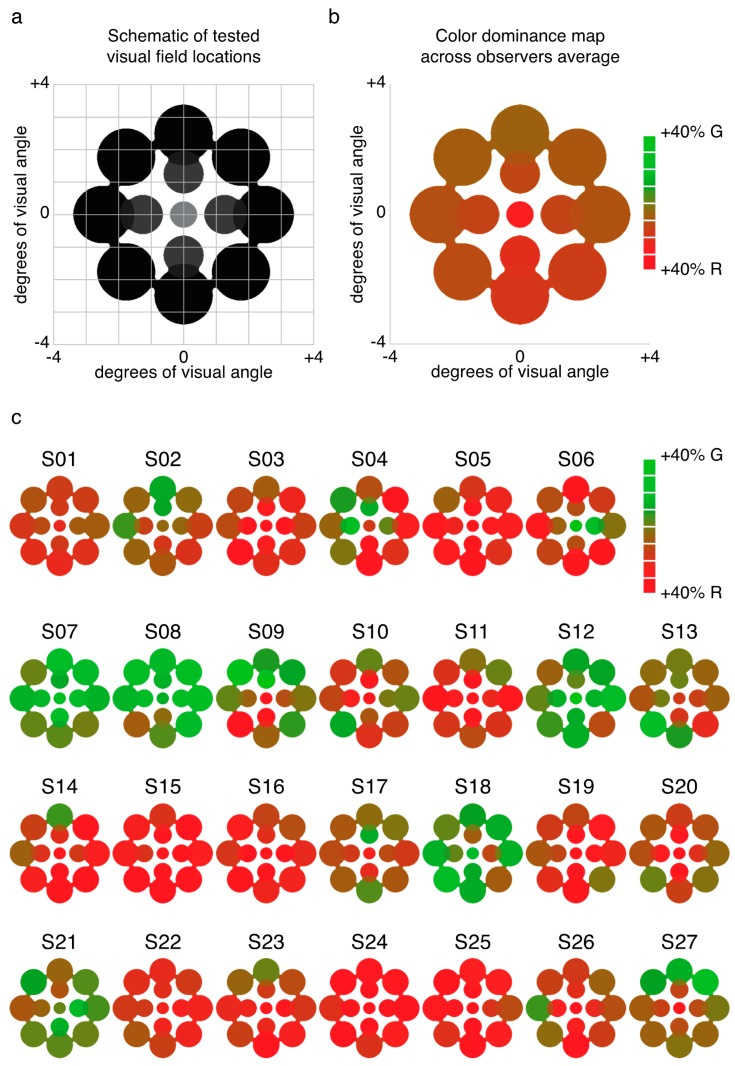
Heat maps of color dominance: (**a**) Schematic illustrating the 13 visual field locations at which rivalry dynamics were tested for each observer (same as Figure 1a, reproduced so this can be viewed as a standalone figure); (**b**) Averaging across all individuals for each location yielded a weak tendency for red dominance that didn’t vary greatly within the visual field. All heat maps are colored so that green indicates green predominance and red indicates red predominance; (**c**) However, individual results indicated unique patterns of color dominance within the visual fields of each observer, analogous to those seen for SED (Figure 1c). For color, though, more observers had biases that affected all tested locations (e.g., see S07 or S24).

The analyses described so far have focused on the impact of eye of presentation while averaging across both stimulus colors (relying on our counterbalanced design). We next investigated the impact of stimulus color by repeating the same analyses, but now averaging across eye of presentation to obtain data on color biases. Results from the bootstrap analysis indicated that across all tested locations, 41/351 (11.7%) of zones showed a color bias that significantly differed from an observer’s overall tendency to report red or green, with 19/27 individuals (70.4%) experiencing at least one significant zone (both analyses at z-score > 2.9 threshold; mean number of significant color zones per observer is 1.5 ± 1.5; Figure 5). At the same time, pooling across all tested locations, more observers experienced a color bias (17/27, 63.0% of observers) than experienced an SED bias (37.0%, see above). Biases for red also appeared to be more common (see Figure 5c), and the across observers’ average also tended towards a red bias (Figure 5b; [33]). Such a “global” bias could result if an observer did not achieve perceptual equiluminance between the colors based on the pre-test flicker fusion experiment.; however, this alone would not explain variability within the visual field as identical luminance values were tested at each location. Note that because we counterbalanced the color-eye pairing across tracking blocks, a zone with strong color bias could not incidentally produce what appeared to be a zone of strong eye dominance. Instead, our experimental design in which these two effects are counterbalanced likely resulted in only the stronger of the two impacts in a particular zone producing a measureable effect in our dataset. Across the metrics described above, zones for SED were about twice as frequent as those for color bias.

We next directly investigated SED independent of color by examining data from a study in which binocular rivalry between greyscale grating images was tracked by 13 observers at two peripheral locations (see Section 4.1.6.). Results from that data set, collected for a different purpose, indicated that SED was not reliably correlated (r = 0.3, *p* = 0.32) across the two locations (one location in each the left and right visual field). The lack of relationship in that data set is consistent with our findings reported above when testing with colored grating stimuli, leading us to conclude that independence in SED within different regions of an observers visual field is not peculiar to color stimuli.

Finally, for each tested location and each observer, we examined the connection between “onset” and prolonged biases by relating the frequency of initial dominance for one eye or color across the 4 tracking blocks to the observer’s overall bias for the second half of the same tracking block (i.e., excluding the first 30 s, to eliminate any potential influence of the first percept on our metric of overall bias). Results revealed a strong relationship between the first perceived eye and an observer’s overall SED (r = 0.35, *p* < 10^−9^). This finding matches previous results from tests in foveal vision [15,25]. Interestingly, we found no relationship between the first perceived color and overall color bias (r = 0.08, *p* = 0.15; see [27]). This pattern across color and eye mirrors the somewhat contradictory findings in the literature, furthering support for a distinction between eye and color with regards to perceptual biases during binocular rivalry (see Section 4.1).

### 2.2. Experiment 2 Results

**Figure 6:**
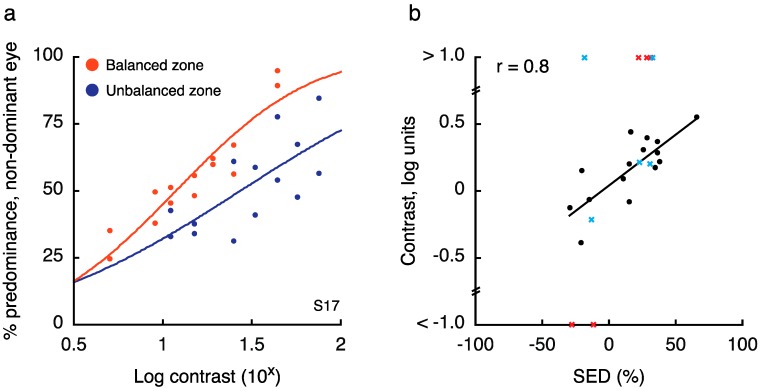
Balance contrast results: (**a**) Results from an illustrative observer. While contrast in this observer’s dominant eye was held fixed (15% contrast), the contrast in the other eye was varied across tracking blocks (x-axis). This change in the physical contrast balance between the two eyes had a systematic influence on the percent predominance of the weaker eye at regions of the visual field where the two eyes were balanced (orange) and unbalanced (purple). In particular, equal predominance (50%) was obtained at a higher physical contrast value at the unbalanced visual field location; (**b**) Across observers, the amount of contrast that had to be added to the weaker eye was strongly related to the SED value. For several observers we obtained impossible contrast values when attempting to fit the data with a cumulative normal function, perhaps due to a strong influence of color in the locations that were tested. Data from the first attempt for these observers are indicated by red X marks on the plot. We retested these individuals using two new visual field locations in a second session (these data are indicated by blue X marks on the plot). Note that two of these observers again yielded impossible values on testing day 2 (blue X’s marked at >1).

In this experiment, we sought to establish how balanced and unbalanced SED regions of the visual field differ in terms of effective contrast. To this end, we measured the amount of contrast that had to be added to the weaker eye at an unbalanced location to equate its predominance with the stronger eye (termed the “balance contrast;” [14]). Figure 6a shows how predominance changed with changes in non-dominant eye contrast for one illustrative observer. Across all tested observers (who had each previously participated in Experiment 1), we found that SED was indeed strongly correlated with the amount of contrast that needed to be added to the weaker eye (Figure 6b; r = 0.8, *p* < 10^−4^). Furthermore, tests at the balanced location yielded a balance contrast close to 15% (15.4 ± 8.1% contrast), indicating a close match to the 15% contrast presented to the “dominant” eye. Critically, a within-observers t-test indicated that there was a significant difference in the balance contrast as measured for the balanced versus unbalanced zone (t_17_ = 5.1, *p* < 10^−4^).

As part of this experimental session, we also investigated observers’ eye movements while they viewed binocular rivalry at the same balanced and unbalanced locations described above. For this test, the contrast of each eye’s image was fixed at 15% as in Experiment 1. First, we found that SED measured under these conditions (i.e., while eye position was tracked, and on a different experimental setup) were closely related to those obtained in Experiment 1 (r = 0.59, *p* = 0.0003). We also found that there was no reliable change in the pattern of eye movements when rivalry was tracked at a balanced versus unbalanced location. Specifically, under both conditions observers generally maintained fixation within ± 0.5 dva of the center of the fixation stimulus (66.1 ± 27.1% of fixations when tracking at the balanced location, 60.1 ± 23.6% of fixations when tracking at the unbalanced location, t_16_ = 0.4, *p* = 0.72). Importantly, fixations of the peripheral rival stimulus accounted for a small percentage of the total tracking time, and this percentage did not differ across tests at the balanced and unbalanced locations (0.00 ± 3.8% and 0.02 ± 1.4% of fixations, respectively, t_16_ = 0.5, *p* = 0.62).

### 2.3. Experiment 3 Results

**Figure 7:**
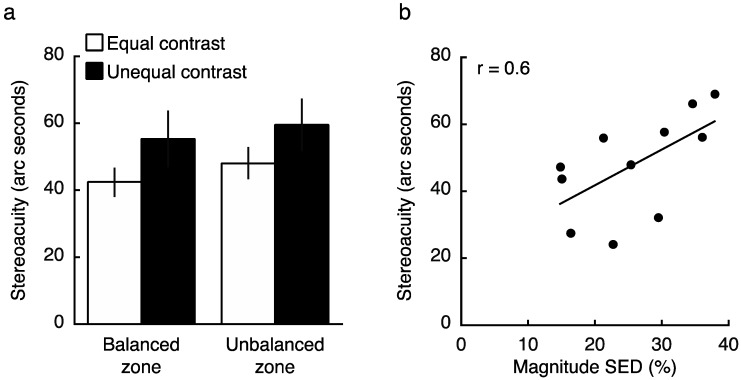
Stereoacuity results: (**a**) We ran a stereoacuity experiment in which observers viewed targets that had physically equal contrast (15%) across the two eyes, or in which the non-dominant eye viewed the balance contrast (see Experiment 2) while the dominant eye continued to view 15% contrast. Consistent with previous results, we found that presenting physically unbalanced contrasts to the two eyes resulted in impaired stereoacuity relative to the equal contrast condition (F_1,10_ = 5.5, *p* = 0.042). Interestingly, this effect was not modulated by the level of SED balance underlying the particular region of visual space in which the test was conducted. (**b**) Considering only the trials on which physically equal contrasts were presented to the two eyes, we found a weak but significant relationship between the magnitude of SED and an observer’s stereoacuity in the visual periphery within unbalanced zones (r = 0.6, *p* = 0.049). This relationship is also significant if both balanced and unbalanced zones are included in the analysis (r = 0.47; *p* = 0.029).

To determine how contrast and SED impacted stereoacuity, we next conducted an experiment in which observers (who all previously completed both Experiments 1 and 2) performed a depth judgement at a balanced or unbalanced region of the visual field, with stereoacuity targets either fixed at equal contrasts in the two eyes (both 15%), or with the non-dominant eye viewing the balance contrast obtained in Experiment 2 (with dominant eye fixed at 15%). Consistent with previous findings [8,9], a Two-Factor repeated measures ANOVA (contrast condition, location) revealed a main effect of contrast on stereoacuity (Figure 7a; F_1,10_ = 5.5, *p* = 0.042). Specifically, when the two eyes viewed physically unmatched contrasts, stereoacuity was impaired relative to the condition in which both eyes viewed equal contrast. Interestingly, SED did not modulate this result, with no main effect of test location (F_1,10_ = 0.8, *p* = 0.40), and no interaction between zone and contrast condition (F_1,10_ = 0.03, *p* = 0.87). Because several outliers were excluded (see Section 4.3), we repeated the analysis including these outliers but normalizing data to each individual’s grand mean stereoacuity, and the results were qualitatively similar (main effect of contrast on stereoacuity; F_1,14_ = 3.5, *p* = 0.077; no main effect of test location; F_1,14_ = 1.5, *p* = 0.24; no interaction; F_1,14_ = 0.001, *p* = 0.98). These results suggest that while presenting a physical contrast balance is sufficient to overcome SED within a binocular rivalry paradigm (Experiment 2), it does not function in the same manner during a stereoacuity task. Despite marked differences between the zones in SED on average (magnitude 4.8% vs. 27.2% at balanced vs. unbalanced locations respectively, t_14_ = 6.5, *p* < 10^−4^), the impact of a physical contrast imbalance was similar (Figure 7a).

Though the average results did not reveal a difference between balanced and unbalanced SED zones in stereoacuity when viewing targets of physically matched contrast (follow up *t*-test excluding outliers: t_10_ = 0.98, *p* = 0.35), we did find a significant correlation between the magnitude of SED and one’s stereoacuity as measured within their unbalanced zone (r = 0.6, *p* = 0.049; including both balanced and unbalanced zones together; r = 0.47, *p* = 0.029; also see [25]). Figure 7b reveals that the difference between these measures likely arises from the wide range of performance levels on the stereoacuity task (even after excluding clear outliers).

### 2.4. Experiment 4 Results

**Figure 8:**
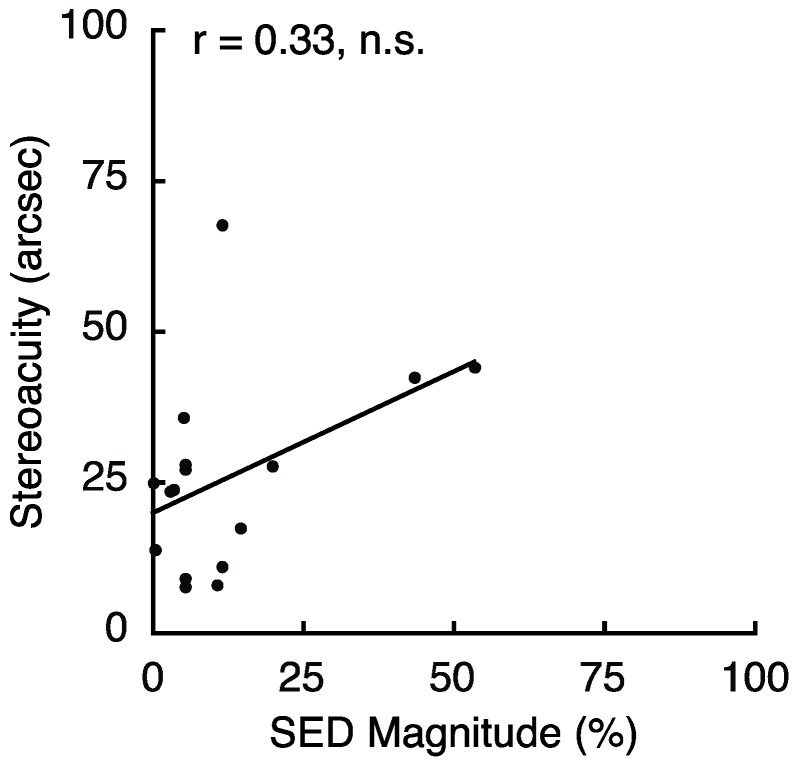
Relationship between SED magnitude and stereoacuity in foveal vision: Because we found that several observers struggled with our stereoacuity task in the periphery, we ran a validation experiment in which we compared the magnitude of SED to stereoacuity in foveal vision. Interestingly, we again found that some observers had very poor performance at this task. Even considering only the observers with relatively good performance, we found no relationship (r = 0.33, *p* = 0.22). The weak statistical power likely results from the low variability in SED magnitude in the sample tested on this study.

To follow up on the relationship between SED and stereoacuity found for peripheral vision in Experiment 3, we conducted an experiment to relate SED and stereoacuity in foveal vision. The overall results revealed no relationship between SED and stereoacuity in foveal vision (Spearman Rank-Order correlation; r = 0.27; *p* = 0.24). However, as in Experiment 3, we found that several observers demonstrated particularly poor performance on the stereoacuity task. Four observers had stereoacuity greater than 120 arc seconds, and each was more than 6 SDs worse than the mean of the other 16 observers (25.8 ± 16 arc seconds). Because these outliers may have been responsible for the null effect, we repeated the analysis excluding these four individuals (Figure 8). This did not change the results (Spearman Rank-Order correlation, r = 0.33, *p* = 0.22); however, it is notable that our sample contained very few observers with large SED. It remains unclear exactly why some individuals performed so poorly on the task. However, we noticed that the 4 outliers made, on average, only 9.5 adjustments per trial, while the other 16 subjects averaged 17.3/trial. Based on the starting disparities and disparity step sizes employed for the stereo test, reaching 0 disparity would require a minimum of between 9–11 discrete disparity adjustments on any given trial. This leads us to wonder whether a lack of patience/motivation for these few individuals may have adversely impacted their task performance.

## 3. Discussion

Our results reveal that prolonged binocular rivalry tracking is characterized by biases of eye and color that persist across extended viewing durations. Interestingly, these results closely match “onset” preferences for SED, but not for color. This seemingly contradictory pattern mimics previous results in the literature [25,27], and suggests that the consistency of biases in rivalry dynamics across brief and prolonged viewing may depend on the stimulus features that are being dichoptically displayed.

Across binocular rivalry tracking blocks, the first eye perceived as dominant by each observer was strongly related to that observer’s overall SED at the location of the visual field being tested. This matches results from foveal testing [15,25], and adds further support for the existence of local “zones” in the context of binocular vision (also see [34,35]). In turn, these idiosyncratic patterns (see Figure 1) are consistent with the idea that binocular rivalry includes neural events transpiring within early stages of visual processing where neurons have relatively small visual receptive fields. At the same time, we recognize that cooperative interactions between spatially local zones of rivalry are likely to exist and promote global spread of dominance [36,37,38,39] that attenuates local anisotropies in SED when larger rival stimuli are viewed.

The relationship between SED and stereoacuity also raises interesting questions about the possibility of a common mechanism underlying rivalrous and typical binocular vision. For example, recent literature has surmised a critical role for normalization in driving interocular suppression [40,41], a mechanism also proposed to be involved in binocular contrast gain control [42,43]. Indeed, the correlation between SED and stereoacuity, with imbalance between the eyes during rivalry associated with impaired binocular vision (Figure 7; [25]), might be consistent with a common mechanism. However, it is interesting that presenting the balance contrast to the weaker eye equalized binocular rivalry dynamics (Experiment 2), but did not improve stereoacuity (Experiment 3). This pattern of results instead suggests that the visual system may already intrinsically correct for SED imbalance when computing depth from disparity. Such a mechanism has been proposed to correct for differences in optical blur between the eyes [44].

Our results also highlight an interesting distinction between color and eye dominance within the context of binocular rivalry tracking. While SED was consistent across both brief and prolonged rivalry viewing, the same was not true for color. This matches results from [27], who found that biases in onset rivalry for one color did not persist when rival targets were viewed for one minute durations. Notably we also found that observers were more likely to have color biases that affected all tested visual locations than was the case for eye biases pooled across all locations. This may suggest that, at least in our study, some observers did not perfectly match the luminance of the red and green items during the equiluminance test, yielding one stimulus that was physically stronger than the other. However, there is also some evidence that red images predominate during binocular rivalry [33], and that observers are less sensitive to flashes of green than red light in the far periphery ([45,46]; but note that these differences are typically found well beyond the eccentricities tested in the present study).

Alternatively, these results may simply point to onset rivalry as a phase of rivalry that is more susceptible to modulation by factors that have no influence on the dynamics of ongoing rivalry. In addition to color and SED (noted above), evidence suggests that attentional modulations of binocular rivalry are quite strong at its onset but quickly wane over time [30]. Recent empirical results have suggested that at least for attentional modulation, this change in efficacy over time may be tied to the level of unresolved perceptual competition between rival images [47]. In fact, if dichoptic images are presented very briefly (for less than 150 ms), observers experience “abnormal fusion” of orthogonal patterns [28,29], a perceptual experience seemingly consistent with a lack of resolution of rivalry conflict.

What, though, would make the susceptibility of binocular rivalry dynamics to factors other than eye wane after this initial period? One idea is that if another mechanism has already taken care of resolving binocular rivalry toward one of the two images, there simply is not anything left for other mechanisms (e.g., attention or feature-specific processing related to the stimuli) to do. Under this scenario, because the factor of eye does persist over time, it suggests that this may be fundamental to the instigation of binocular rivalry, consistent with many previous results [20,21,22,23,24]. In support of this idea, recent evidence from perceptual learning paradigms indicates that the bulk of the changes to rivalry dynamics following training are specific to the trained eye rather than stimulus [19,48]. Taken together, our results do support the idea that “onset” rivalry is partially distinct from ongoing binocular rivalry dynamics [27], in that it is more susceptible to a larger host of factors that can influence perception than is prolonged viewing.

Finally, these results pose a methodological issue for studies that involve imaging rival targets at multiple different locations of the visual field. In particular, studies of both binocular rivalry and the closely related paradigm of continuous flash suppression (CFS, [49]) often involve comparing duration data collected within distinct regions of the visual field. Our results indicate that these regions are likely to have separable biases across at least two dimensions if rivalry stimuli are briefly presented, with potentially large biases in eye dominance persisting if stimuli are viewed for long periods of time. Adding to this, variability in the visual field for other aspects of spatial vision (e.g., crowding) has recently been reported [50]. Researchers utilizing this experimental setup will want to obtain baseline measurements of SED and feature biases within the regions of the visual field being tested, to ensure that this confound does not impact the variables of interest.

## 4. Materials and Methods

Experiments 1–3 were designed together as parts of a common study based on results from a pilot study [51]. Our goal was to measure variability in SED and color dominance within the visual field of multiple observers (Experiment 1), and then to follow up on these measurements by testing the balance contrast (Experiment 2) and stereoacuity (Experiment 3) within zones that differed in SED for each individual. Importantly, this means that the design of Experiments 1-3 was established before we had any knowledge of the results.

In line with this plan, we recruited volunteers who stated that they were willing to complete all three experiments. However, as outlined in the specific methods section for each experiment, some observers voluntarily withdrew before completing all experiments. Experiment 4 was added as a follow-up experiment based on our Experiment 3 results, and we recruited a new sample of observers for this study.

### 4.1. Experiment 1 Method

**Figure A1:**
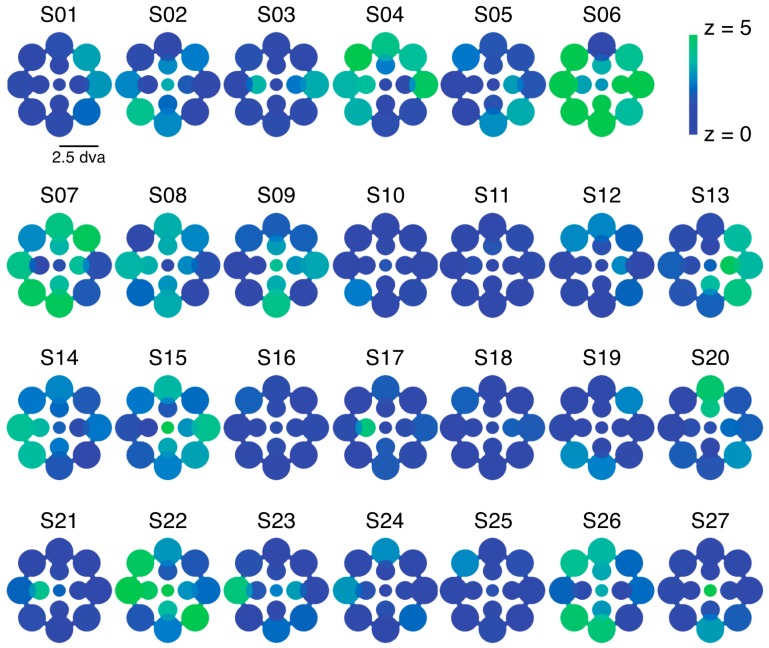
Statistical reliability maps for all individuals, eye dominance: Heat maps of results from our bootstrap test to estimate the statistical reliability of each zone of the visual field. In this analysis, we compute a z-score based on bootstrap samples that indicates the likelihood of observing an SED value in that zone as extreme as the one we did observe, given that individual’s overall eye bias when collapsing across all zones. Here we include z-score maps for all 27 individuals in Experiment 1 (note that S23 is the same as S23 in Figure 2a; figure corresponds to raw values in Figure 1 and Table A1).

**Figure A2:**
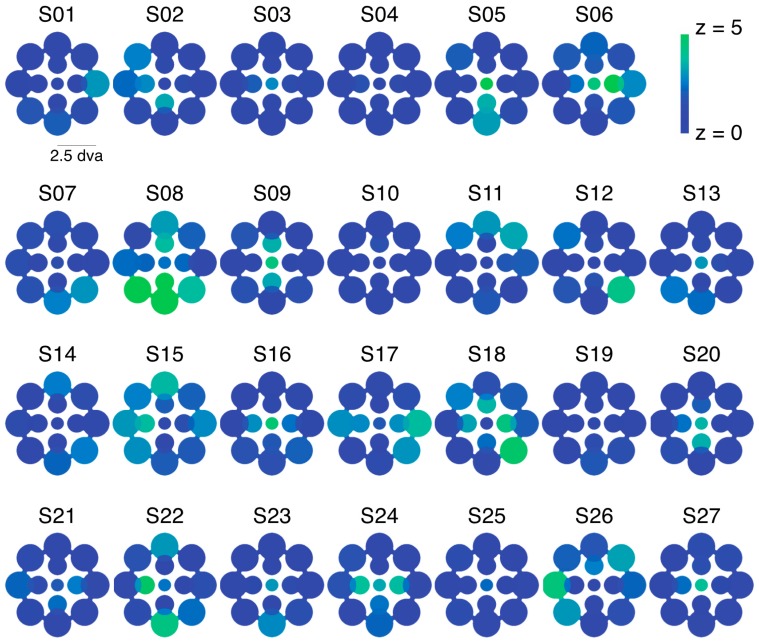
Statistical reliability maps for all individuals, color dominance: Heat maps of results from our bootstrap test to estimate the statistical reliability of each zone of the visual field. In this analysis, we compute a z-score based on bootstrap samples that indicates the likelihood of observing an color dominance value in that zone as extreme as the one we did observe, given that individual’s overall eye bias when collapsing across all zones. Here we include z-score maps for color dominance for all 27 individuals in Experiment 1 (corresponding to raw values in Figure 5 and Table A2).

Experiment 1 was designed to measure patterns of eye and color predominance across 13 visual field locations for each observer. While viewing dichoptically presented, colored, rotating gratings at one location during each tracking block, observers used key presses to indicate the successive durations of exclusive visibility of one rival target or the other. The resulting data were used to produce color-coded “heat maps” (see Figure 1, Figure 2, Figure 5, Figure A1 and Figure A2) portraying the spatial distribution of eye and color dominance within the visual field.

#### 4.1.1. Observers

27 observers (15 females, 12 males; median age 20 ± 7.9 years) participated in Experiment 1. These volunteers were recruited through advertisements posted in the Vanderbilt University Psychology Sign-Up System with the intention that they would complete Experiments 1–3 (in that order). All 27 participants agreed to participate in all sessions for Experiments 1–3; however, as outlined before each experiment, some voluntarily withdrew before completing all experiments. One observer (KD, S21) is also an author; the other observers had (to our knowledge) little or no prior experience viewing binocular rivalry, and they were naïve as to the purpose of this study. Laboratory testing confirmed that each individual had monocular and binocular acuity of 30/20 or better. Although there was some variability in foveal stereoacuity (Randot^®^ Stereotest, Stereo Optical Co., Inc., Chicago, IL, USA, 2004), each observer had stereoacuity of 70 arc seconds or better (mean 37.9 ± 15.9 arc seconds). Note that Experiments 3 and 4 directly investigated individual variability in peripheral and foveal stereoacuity. The study was conducted in accordance with the Declaration of Helsinki. All procedures were approved by the Vanderbilt University Human Research Protection Program (IRB #010110), and each observer provided written informed consent prior to participation.

#### 4.1.2. Apparatus

Stimuli were generated using the MATLAB Psychophysics Toolbox (MATLAB 2011a, MathWorks, Natick, MA, USA) [52]. They were presented on a linearized Sony GDM-FW900 monitor (Sony, Tokyo, Japan) (1024 × 768 resolution) running at 100 Hz. Observers viewed stimuli through a mirror stereoscope that was mounted on a chin rest, fixed 92 cm from the display (viewing distance through the mirrors).

#### 4.1.3. Stimuli

During 60-s tracking blocks, observers experienced binocular rivalry by viewing dichoptically presented orthogonal sine wave gratings (15% Michelson contrast created using Gamma corrected monitor luminance values based on measurements from a Minolta LS-110 photometer (Minolta, Tokyo, Japan). These gratings synchronously rotated clockwise at 0.25 rot/s (so that the orientations in the two eyes varied continuously while always remaining orthogonal). The two gratings differed in color to permit our analyses of this rival feature, with this color difference (one grating was red, one was green) also permitting easy identification of rivalry predominance. The two colors were matched for luminance based on a preliminary binocular flicker-fusion equiluminance test completed by each observer. All observers completed a rivalry tracking simulation during a practice session that ensured each observer was able to accurately distinguish between the two colors.

Across 26 blocks, observers tracked rivalry alternations at 13 different visual field locations (repeated 2 times each, with color-to-eye assignment counterbalanced across the two runs). One location was at fixation, four were centered at an eccentricity of 1.25 dva, and eight were centered at an eccentricity of 2.5 dva from fixation (see Figure 1a). Rival images were scaled with eccentricity for size (1, 1.4, and 1.96 dva) and spatial frequency (3, 2.1, and 1.5 cyc/deg). On all runs, rival images were surrounded by a white circle to facilitate alignment of the rival images (also see details of alignment procedure below). For all peripheral runs, a fusible image at fixation was presented (for tests at fixation, a small white fixation point was presented at the stimulus center).

#### 4.1.4. Procedure

Experiment 1 consisted of one practice session and two main testing sessions. The practice session (~30 min) could be completed on the same day as the first main testing session (15/27 participants did so), but the two main testing sessions were always conducted on separate days.

At the start of the practice session, each observer completed 10 trials of a flicker-fusion equiluminance test (stimuli presented binocularly through the stereoscope), and performed a custom alignment procedure in which they used key presses to move left eye (LE) and right eye (RE) fusion stimuli on the screen until they were properly aligned. This process was completed three times, with the average LE and RE image coordinates then used to position the central fixation image during all rivalry blocks. For blocks where rival stimuli were presented away from the fovea, observers completed another alignment just before beginning the tracking period. Here, while maintaining fixation on the centrally presented fixation image, an observer used key presses to align a flashing peripheral fusion image presented to his/her RE while a static, identical image was presented to the LE.

After these calibration procedures were complete, observers practiced binocular rivalry tracking while eye position was monitored online (via an EyeLink 1000 desktop mount that viewed each observer’s LE through the stereoscope setup). For each tracking block, observers pressed and held down keys on the keyboard (left and right arrow) to indicate sustained dominance of the green or red image (respectively). They released both keys to indicate a mixed percept. Auditory feedback was given any time eye position drifted more than 1 degree from the fixation point (evaluated at 0.5 HZ). This relatively large fixation window was necessary in the practice session because naturally occurring linear drift in eye position during the 60-s tracking block could not be corrected online. However, pilot testing revealed that this procedure was sensitive enough to detect direct fixations of the peripheral stimuli. Five tracking blocks were tested during the practice session:

*Practice* *Block* *1:* Simulation of binocular rivalry at fixation.
*Practice* *Block* *2:* Real rivalry at fixation.
*Practice* *Block* *3:* Simulation of rivalry in periphery (1.25 dva eccentricity).
*Practice* *Block* *4:* Real rivalry in periphery (1.25 dva eccentricity).
*Practice* *Block* *5:* Real rivalry in periphery (2.5 dva eccentricity).

Simulations of binocular rivalry were produced by physically alternating stimulus presentations between monocular LE and RE images (stimuli identical to those used on real rivalry runs). Percept durations were predetermined at random from a gamma distribution with a mean of 3 s. These trials also included simulated mixed percepts, which were produced by generating a movie that transitioned smoothly from one image to the next. To produce simulated mixtures, we divided the stimulus into a grid of 9 blurred squares and linearly transitioned each grid element from one to the other stimulus over a random period of time (max duration of 2 or 3 s across different simulated mixtures). Pilot testing revealed that presenting these movies binocularly produced adequately realistic mixtures, i.e., the to-be-dominant eye viewed a movie that smoothly transitioned from a grey background to the next dominant image, while the previously visible eye viewed a movie that smoothly transitioned from one rival image to the next (e.g., green to red grating). After each tracking period of simulated rivalry, the experimenter visually inspected a plot showing times of stimulus changes and key presses to confirm that each observer produced accurate key presses while viewing the simulation. On real rivalry blocks, orthogonal rotating gratings of different colors were presented to the two eyes as in the main experiment. For practice blocks, color-eye assignment and peripheral location (for blocks 3–5) were chosen at random from the set of possible locations (see Figure 1a).

Observers completed the equiluminance test again at the start of the first main testing session, and completed the image alignment procedure at the start of each session. As in the practice trials, observers also completed a peripheral alignment procedure immediately prior to each tracking block for the location that was about to be tested. On each tracking block, observers used key presses (as above) to indicate predominance of the green or red image, and released both keys to indicate a mixed percept. Each rivalry tracking period lasted for at least 60-s, but continued beyond 60-s until the observer’s next change in key press to ensure the final percept was not truncated. Both main testing sessions consisted of 26 tracking blocks, with color-to-eye assignment counterbalanced across the 13 tested locations within each session. The order in which the locations were tested was randomized independently for each observer in each session.

#### 4.1.5. Data Analysis

During the tracking experiment, observers pressed (and held down) the left or right arrow key on the keyboard to indicate exclusive perceptual dominance of the green or red (respectively) rotating grating. When mixed percepts were experienced, the observer released both keys. For all analyses, only key presses/releases lasting longer than 300 ms were included, as shorter durations more likely reflect errant key presses than true visual percepts. To compute the relative predominance of each eye during binocular rivalry, we computed a metric based on the proportion of time that an observer reported each eye as exclusively dominant at each location [15]:

SED = (RE_prop_ − LE_prop_)/(RE_prop_ + LE_prop_) × 100 (1)

This gave us a quantitative assessment of sensory eye dominance (SED). For the main analyses, we pooled tracking data across both sessions (4 blocks; Figure 1, Figure 2, Figure 4, Figure 5, Figure A1 and Figure A2). However, for test/re-test analyses (Figure 3), we pooled data separately for each session.

To produce heat maps (Figure 1, Figure 2, Figure 5, Figure A1 and Figure A2), we plotted 13 disks (using fspecial in MATLAB) centered on each test location, with size scaled to match that used in the experiment at each location. The magnitude of each was scaled based on the observed SED, z-score, or color dominance value and sign. Small portions of the visual field were covered by two test locations (see Figure 1a and Figure 5a); in these regions of the heat maps, we plotted the average SED from the overlapping stimulus locations. These heat maps were colored in MATLAB using imagesc and then cropped and resized (to remove portions of the map with no data) in Adobe Photoshop using an automatic batch editing procedure. To reiterate, the automated editing procedure was identical for all heat map images and only involved cropping and resizing (Figure 1, Figure 2, Figure 5, Figure A1 and Figure A2).

To investigate the observed variability across local regions of the visual field (Figure 2), we employed a resampling procedure to estimate the likelihood that results occurred due to chance. First, we investigated whether “zones” of eye dominance (i.e., SED within any local patch) truly differed from the pooled eye dominance (i.e., average SED across all tested locations) for a given observer. For this analysis, we pooled all LE and RE durations across all tested locations for each observer. For each of the 13 tested locations, we then drew 10,000 bootstrap samples (randomly with replacement) that were matched in the number of LE and RE percepts to the observed values [16]. Based on the mean and standard deviation of these bootstrap samples, we next computed a z-score for each observed value (see Figure 2a). Note that this analysis establishes the relative SED of each zone for a given observer, but does not tell us about the absolute level of SED. In other words, a zone with equal balance between the eyes could have a high z-score if an observer’s overall (i.e., spatially pooled) eye dominance is unbalanced.

We next conducted a similar analysis to establish whether an observer’s spatially pooled SED differed from zero (equal balance). For this analysis, we created one pool of all observed durations for an observer (across location and eye). We then drew 10,000 bootstrap samples (randomly, with replacement) matched to the overall number of total percepts for that observer, and produced a “global” z-score for each observer (as above). Analogous simulations were conducted to investigate the impact of color on our results, by fixing the number of “red” and “green” percepts in place of LE and RE.

We then investigated the test/re-test reliability of our results in three ways. For all methods, we first computed SED separately for each location and testing session. In the first analysis, we computed the absolute value of the difference between observed SED values from session 1 and 2, taking the median difference value for each observer across the 13 locations as a measure of reliability (Figure 3a). As a second method, we pooled all individual location data points from day 1, and plotted them against day 2 results (Figure 3b). Finally, we implemented a multilevel linear mixed effects model in SAS (code available through figshare: [53]). Details are provided in the main results.

Relying on results from a recent study in our lab, we also looked for signatures of binocular rivalry dynamics that are characteristic of imbalances in SED [15]. First, we computed the difference in median duration between an observer’s LE and RE percepts within a given zone of the visual field (Figure 4a). Here, the median percept duration for each eye was computed by combining data across all 4 tracking blocks at each tested visual field location. We also identified transitions in our data simply by noting instances where observers switched their key press (with out without an intervening mixed percept). Using this data, we investigated the frequency of return transitions (FRT) in our data [32], which were defined as any instance where an observer indicated subsequent percepts from the same eye with an intervening mixed percept. To compute the overall FRT, we simply divided the number of these occurrences by the total number of transitions overall. We also calculated frequency for each eye separately, and produced a combined metric that indicated the relative FRT to each eye (Figure 4b; after [15]):

FRT_REL_ = (FRT_RE_ − FRT_LE_)/FRT_TOT_ × 100 (2)

Note that for zones that had no return transitions (and thus a denominator of 0), the value of FRT_REL_ was set to zero (as there was indeed no difference between return transitions to the LE and RE).

Finally, we compared the dynamics of “onset” rivalry to long-term rivalry tracking by correlating the proportion of trials that an observer’s first reported exclusive percept was the left eye (or alternatively, green stimulus) with the overall SED (or color dominance) within that visual field zone during the latter half of the trial (i.e., excluding the first 30-s, to ensure the impact of the first percept was not included). For each tracking block, the first percept was defined as the first key press made by the observer to indicate exclusive predominance of the red or green image. We then correlated the proportion of individual tracking blocks for each visual field location (out of the total of 4) for which the left eye, or green stimulus, was dominant. These proportions were compared to SED and color bias, respectively, for the entire tracking block, in order to establish the correlation between initial and prolonged rivalry biases.

#### 4.1.6. SED with Greyscale Stimuli

Because we found that observers had local ”zones” of both eye and color dominance, we wanted to examine whether the SED results required the use of colored stimuli. We utilized a dataset from a separate project in the lab in which 13 observers (6 male, 7 female, age 22 ± 5 years) viewed binocular rivalry between greyscale grating images (1.5 dva diameter, 40% contrast, 2.67 cyc/deg) at two peripheral locations (1.8 degrees eccentricity). To minimize adaptation this study utilized dynamic gratings that rotated within a 70 degree range (10–80 or 100–170; each rotational episode completed in 1 s). The orientations in the two eyes were always orthogonal, and the restricted rotation range ensured that one was always tilted categorically to the left, and the other to the right (to enable perceptual reports). The eye-to-tilt pairing was counterbalanced across 24 one-minute tracking blocks at each location, tested across two sessions on separate days. We calculated SED as described above for these images, and correlated the results at each location to see if SED at one location was predictive of SED at the other location.

### 4.2. Experiment 2 Method

Previous results have quantified SED in terms of an effective contrast deficit by measuring the amount of contrast that needed to be added to the weaker eye in order to achieve equal predominance with the stronger eye [14,16]. We sought to replicate these results by testing whether the balance contrast differed within the visual field of each individual observer by directly comparing results in one balanced zone (small magnitude SED) and one unbalanced zone (large magnitude SED). As discussed above, because this experiment was designed and run prior to the completion of Experiment 1, we classified each zone as “balanced” or “unbalanced” using criteria based on our pilot results ([51]; see Section 4.2.4. below for full details). As part of this experimental session (but separate from the balance contrast measurements), we monitored observers’ eye position while they tracked peripheral rival stimuli to ensure that Experiment 1 results were not due to unique patterns of eye movements depending on the tested visual field location.

#### 4.2.1. Observers

22 observers (12 females, 10 males; median age 20 ± 4.3 years) participated in this study. All observers had previously participated in Experiment 1. Three observers who completed Experiment 1 withdrew before participating in Experiment 2. In addition, the two observers with very poor test/re-test reliability (>4 SDs less reliable than mean of the other 23 observers across sessions) did not complete Experiment 2 (see Section 2.1). Because the aim of this experiment was to compare the balance contrast within observers at one balanced versus one unbalanced SED zone (see below), one observer (S25) who had essentially no variability in SED across zones did not participate in the balance contrast measurement. He was the only observer who, at both eccentricities, had no two zones that differed by at least 10% in SED magnitude. He still completed the eye tracking experiment to ensure that eye movements did not account for this low SED variability; however, these data were considered separately from those of other observers where the goal was to compare patterns of eye movements across the balanced and unbalanced locations. Data from one additional observer (S12) were excluded from analyses due to an experimenter error that was not discovered in time to retest this individual. For the eye tracking analysis comparing balanced to unbalanced test locations, S25 and S12 were excluded for the reasons just described, and S05 could not be tested due to an inability to complete the eye tracker calibration procedure.

#### 4.2.2. Apparatus

The apparatus for the balance contrast experiment was identical to that used in Experiment 1. For the eye tracking experiment, a similar setup in a separate testing room was used. This setup utilized a linearized Sony CPD-E540 monitor (Sony, Tokyo, Japan) (1024 × 768 resolution) running at 100 Hz. Viewing distance for this setup was 80.5 cm through the mirrors. Eye tracking was conducted using an EyeLink 1000 desk mount (SR Research, Ottawa, ON, Canada) that could view each observer’s left eye through the mirror stereoscope.

#### 4.2.3. Stimuli

Observers viewed orthogonal, rotating sine wave gratings as in Experiment 1. During the balance contrast and eye tracking experiments, binocular rivalry stimuli alternated across blocks between 2 of the 13 locations tested in Experiment 1 (see below for selection procedure). In the balance contrast experiment the contrast presented to one eye (the non-dominant eye) was varied across 7 levels while contrast of the grating viewed by the other (dominant) eye was held constant at 15%. During the eye tracking experiment, contrast was fixed at 15% in both eyes during all tracking blocks. As in Experiment 1, the color-to-eye pairing was counterbalanced across tested locations and contrast levels.

#### 4.2.4. Procedure

Prior to each individual’s participation in Experiment 2, the experimenter (author KD) selected two regions of interest for that observer for further testing. The goal was to conduct follow up tests in one balanced region of an observer’s visual field (i.e., lowest possible SED) and one unbalanced region (i.e., magnitude of SED as large as possible; required to be >10%). Note again that these criteria were established before any results were obtained from Experiment 1, based on pilot data [51]. The idiosyncratic pattern of results we observed in that pilot study (as in Figure 1) necessitated an individualized approach. However, some general rules were followed:

- Both test locations were always at the same eccentricity
- Observed SED magnitude in the balanced zone must not exceed 10%
- Observed SED magnitude in the unbalanced zone must exceed 10%

While the goal was to choose the smallest and largest SED magnitude observed for each individual, the experimenter also considered the reliability of the results for each zone across the 4 repeated tests in Experiment 1. The primary goal of this selection procedure was to quantify SED in terms of contrast so that its impact on stereoacuity could be tested in Experiment 3 (see below). Therefore, zones where SED varied widely across tracking blocks were avoided.

During the balance contrast experiment, observers alternately viewed binocular rivalry at the two chosen locations. Across tracking blocks, the contrast of the image presented to the observer’s non-dominant eye (defined as the less predominant eye, based on Experiment 1 data, at the unbalanced test location) was systematically varied in contrast (values presented in a randomized order). Non-dominant eye contrast varied across 7 levels (between 5–75%, presented in a randomized order), while dominant eye contrast was fixed at 15%. Identical contrast values (5, 9, 11, 15, 19, 25, and 44%) were always presented at the balanced location for all observers. The specific contrast values presented at the unbalanced location were selected by the experimenter from a fixed set of 11 contrast values (ranging from 5–75%), depending on SED. While there was a degree of subjectivity in these decisions, the experimenter relied on pilot results ([51]), as well as published findings [14,16] regarding the shift in balance contrast for various SED magnitudes to make these determinations. The equal contrast condition was always tested, and the experimenter aimed to pick a sufficiently wide range of contrast values that spanned both sides of the 50/50 predominance point for each observer. Establishing the balance contrast involved fitting the resulting data with a cumulative normal function (see below), a process that is robust to differences in the tested contrast conditions. All other aspects of this experiment were identical to Experiment 1.

Immediately following the balance contrast experiment, observers completed the brief eye tracking experiment. Here, observers viewed binocular rivalry in 4 tracking blocks that alternated between the two testing locations from the balance contrast experiment. For this experiment, contrast was fixed at 15% in each eye. Observers completed a brief eye tracker calibration prior to each tracking block, and eye position was monitored throughout each 60-s tracking period. Unlike the practice session, no auditory feedback was given during the block.

#### 4.2.5. Data Analysis

The goal of our analysis was to establish the balance contrast for the observers in this experiment—that is, the amount of contrast that needed to be added to the non-dominant eye in order for it to achieve equal predominance with the dominant eye [14,25]. We first computed the proportion predominance for the non-dominant eye for each block at each location. This data was fitted with a cumulative normal function (using fminsearch in MATLAB), with the point at which this function passed through a predominance of 0.5 taken as the balance contrast for that observer at that tested location [16].

Note that for 5 observers, this procedure resulted in impossible balance contrast estimates (i.e., estimated values greater than 100% contrast). Each of these observers was retested on this experiment with new locations chosen for run 2 (run 1 results for retested individuals indicated as red X’s in Figure 5b), as a key goal of this experiment was to obtain a usable value of balance contrast for Experiment 3. For 3/5 retested observers, a usable value was obtained on run 2 (run 2 results for retested individuals indicated as blue X’s in Figure 5b). It is possible that color drove predominance considerably more than contrast in these zones, as predominance data were noticeably unaffected by changes in contrast. To calculate the correlation between SED and balance contrast (Figure 5b), we included observers for whom we obtained a usable value, and excluded the two others for whom we could not obtain an estimate (yielding N = 18 in the final correlation and *t*-test).

For eye tracking data, we were interested in the stability of fixation as well as an observer’s tendency to break fixation to glance at the peripheral stimulus. First, we preprocessed the eye tracking results by removing linear drift along the x and y-axis, and by median filtering the data with a 100 ms sliding window. We then defined a stimulus window (based on the location and size of the presented rival image in the left eye) and a fixation window (1 × 1 dva square centered on fixation). Note that this fixation window is small enough that it excludes the stimulus edge even for the closest eccentricity. To ensure this analysis procedure was effective, we tested it using pilot data sets in which an observer intentionally fixated the rival image for half of the tracking block, and found that this was clearly detected in the output. Thus, these parameters allow us to effectively identify fixations on the peripheral stimuli during tracking blocks.

### 4.3. Experiment 3 Method

In Experiment 3, we sought to relate SED as indexed by binocular rivalry tracking to stereoacuity. Furthermore, we tested whether adding the balance contrast (as measured in Experiment 2) to the weaker eye could actually improve stereoacuity at the unbalanced location by overcoming an observer’s intrinsic eye imbalance. These tests were conducted within the same visual field regions tested in Experiment 2 for each observer.

#### 4.3.1. Observers

15 observers (9 females, 6 males; median age 20 ± 4.6 years) participated in this study. All observers had previously participated in Experiments 1 and 2. Of the 20 observers tested in the balance contrast analysis from Experiment 2, two were excluded because no estimate of balance contrast could be obtained (see Experiment 2 Results). Two were excluded because their balance contrast values at the unbalanced location were very small (within 5% of equal balance; S15 and S16), meaning the equal and unequal contrast conditions in this experiment would be virtually identical and therefore uninformative regarding our hypothesis. One additional observer (S20) was excluded due to unreliability across experiments—Experiment 1 indicated LE dominance at her unbalanced location, while Experiment 2 indicated RE dominance, meaning that interpreting results from this observer would not have been possible. However, recall that reliability across Experiments 1 and 2 was strong overall (Figure 6; note that S20’s data is included in Figure 6).

#### 4.3.2. Apparatus

The apparatus for Experiment 3 was the same one used in Experiment 1.

#### 4.3.3. Stimuli

In Experiment 3, observers viewed fusible images in the LE and RE, consisting of a central grey dot surrounded by a grey annulus. For each observer, the same locations tested in Experiment 2 were tested again in Experiment 3. The surrounding annulus was fixed at zero disparity across the two eyes, while observers used key presses to adjust the relative disparity of the central dot. Depending on which eccentricity was tested, the outer ring either had 1.36 dva diameter with 0.09 dva width, or 1.9 dva diameter with 0.13 dva width, approximately matching the size and scale factor of rival images. The central dot had 0.54 or 0.76 dva diameter, with Gaussian blurring (which allowed sub-pixel rendering) applied to its circumference beyond that radius with SD of 0.04 or 0.06 dva. The starting disparity on each trial was picked randomly from 6 possible values, ±135, ±150, or ±165 arc seconds, and observers adjusted disparity in steps of 15 arc seconds. In the dominant eye, both the central dot and annulus were fixed at 15% contrast across all conditions. In the non-dominant eye, the contrast of both the central dot and annulus were also fixed at 15% contrast in the “equal” condition, and were fixed at the balance contrast for that observer (obtained from Experiment 2) in the “unequal” condition at both locations. On all trials, the stimulus appeared (on for 1.5 s) and disappeared (off for 0.5) to minimize adaptation effects.

#### 4.3.4. Procedure

Observers completed a total of 100 experimental trials, preceded by 24 practice trials. The tested location in the visual field alternated between the two locations chosen for each observer prior to Experiment 2; one location where SED was balanced (close to 0), and one where SED was unbalanced (magnitude greater than 10%), with the test location for the first trial randomly determined. For each location, two contrast conditions were presented in a counterbalanced fashion. In one condition, the physical contrast was fixed at 15% in both eyes (“equal” contrast condition), while in another, contrast was fixed at 15% in the dominant eye (i.e., the eye that was dominant at that observer’s unbalanced location) and at the balance contrast value (from Experiment 2) for the non-dominant eye (i.e., the non-dominant eye at that observer’s unbalanced condition). Importantly, the same physical contrast conditions were presented at both the balanced and unbalanced location of the visual field in order to test the impact of SED on stereoacuity.

Each trial began with the binocular presentation of a central grey dot and surrounding grey annulus, both fixed at a particular contrast on each given trial as outlined above. Observers adjusted the disparity of the central dot by tapping the up or down arrow key, with the goal of matching the perceived depth of the central dot to that of the surrounding annulus. Adjustments could continue for as long as desired, with observers hitting the enter key to finalize their response. Observers were instructed to maintain fixation on a central fusion stimulus for the entirety of a trial, and told not to look directly at the peripheral stimuli. The experimenter emphasized that their entered response should reflect the perceived disparity of the dot while maintaining central fixation, as perception could vary if an observer moved his or her eyes during a trial. The maximum disparity allowed by the code was ±300 arc seconds—if observers attempted to adjust the stimulus beyond this disparity, a tone sounded indicating that they could not move any farther in that direction. All trials on which an observer hit this “wall” were excluded, as this could potentially serve as a cue to where zero disparity was (overall 3.1% of trials were excluded). The four observers with particularly poor performance on this task (see below) accounted for 43 of the 47 total excluded trials (91.5%) across all observers.

#### 4.3.5. Data Analysis

Stereoacuity was computed for each condition by taking the standard deviation of all entered disparity values for a given condition. However, the obtained raw data revealed considerable individual variability, likely owing to the difficulty of making stereo judgments in the visual periphery. To ensure that our results were not obscured by this variability, we conducted two related analyses. In each, we used an observer’s “grand mean” stereoacuity (i.e., as obtained by collapsing across all trial types) as a benchmark for their overall task performance.

In the first analysis (Figure 7), we calculated raw stereoacuity but excluded four observers with particularly poor performance (each of these observers had a “grand mean” stereoacuity of >139 arc seconds, more than 5 SDs from the mean of the other observers which was 57.7 ± 16.3 arc seconds). Figure 7b shows the average raw data for the other 11 observers. In a second analysis, we ensured that excluding this relatively large proportion of our observers did not systematically bias the results. Here, we included data from all 15 observers, but normalized each individual’s stereoacuity in each tested condition to his or her “grand mean” stereoacuity. As detailed in the results, this analysis yielded similar results to those obtained when outliers were excluded, suggesting that these individuals’ poor overall performance overall did not relate to the observed contrast effect.

### 4.4. Experiment 4 Method

In Experiment 3 we found a significant relationship between SED and stereoacuity in peripheral vision. Though this result is consistent with previous findings [25], we wanted to validate our experimental design by measuring SED and stereoacuity in foveal vision. This should reduce the task difficulty present in Experiment 3 (which required peripheral stereoacuity judgments). In addition, previous findings by [25] suggest that the relationship between SED and stereoacuity may be stronger in foveal vision (compare Figure 8a,b in that paper).

#### 4.4.1. Observers

20 observers (13 females, 7 males; median age 21 ± 5.1 years) participated in Experiment 4. One observer was an author (KD) who also participated in Experiments 1–3. All other observers only participated in Experiment 4, and were naïve as to the purpose of the study. These individuals were recruited from the Vanderbilt Psychology Department subject pool, gave written informed consent prior to participating, and were paid for their time.

#### 4.4.2. Apparatus

The apparatus for Experiment 4 was the same one used in Experiment 1 and 3.

#### 4.4.3. Stimuli

Stimuli for the stereoacuity task were similar to those described for Experiment 3 except that they were smaller (inner dot 0.38 dva diameter plus 0.03 dva Gaussian blur beyond that circumference; outer ring 0.98 dva diameter, 0.06 dva wide) and presented foveally. In addition, they were presented at equal contrast (30%) in both eyes on all trials.

During the binocular rivalry tracking blocks, observers viewed orthogonal (±45 degree) sine wave gratings at 30% contrast. Rival targets were 1.5 dva in diameter with a spatial frequency of 2.67 cyc/deg. These gratings were grey-scale, and were surrounded by a fusion image that was identical in both eyes.

#### 4.4.4. Procedure

The binocular rivalry tracking and stereoacuity tasks were completed within a single 30-min session, with the task order counterbalanced across observers. As with the other experiments, observers began by completing a calibration procedure to determine the screen coordinates to properly align the images displayed to the left and right eye.

During the binocular rivalry tracking task, observers viewed orthogonal sine wave gratings for 60-s periods while reporting what they saw using key presses. They pressed and held the left or right arrow key to indicate complete dominance of the left or right tilted grating, and released both keys to indicate a mixed percept. The tracking block continued beyond 60-s until the next change in key press to ensure that the final percept of each block was not truncated. Observers completed a single one-minute tracking block for practice before beginning the main blocks, and were given a mandatory 5 s break between tracking blocks (and could rest for as long as they wished before moving to the next block).

The stereoacuity task began with 30 practice trials followed by 50 main trials (there was no difference between these trials except that observers were explicitly told the first 30 were practice). All other procedural details were identical to the task reported in Experiment 3 except that the stereoacuity target was fixated.

#### 4.4.5. Data analysis

For each observer, we calculated SED for each individual tracking block and then averaged across these blocks for an overall estimate. Stereoacuity was computed by taking the standard deviation of the 50 judgments during the main trials. These results were then compared across observers to yield Figure 8.

## Appendix A

**Table A1:** This table contains the mean and standard deviation SED values corresponding to the individual heat maps in Figure 1. To compute standard deviation, we drew 10,000 bootstrap samples of left eye (LE) and right eye (RE) durations from each zone’s LE and RE data pool for each individual observer. The number of data points in the LE and RE samples were always matched to the observed number of LE and RE data points in each zone [16]. In the table below, eccentricity (ecc) is in degrees of visual angle. The direction (dir) refers to the position of the rival patches relative to fixation, with 0 directly to the left of fixation, and increasing numbers moving clockwise around a circle. In other words, 0 corresponds to West on a compass, 90 to North, etc. Positive numbers correspond to RE dominance; negative numbers to LE.

**Location (ecc, dir)**	**S01**	**S02**	**S03**	**S04**	**S05**
0, n/a	1.1 ± 11.5%	26.8 ± 7.4%	−40.0 ± 9.7%	47.0 ± 8.4%	7.9 ± 22.0%
1.25, 0	16.3 ± 6.7%	33.9 ± 5.5%	−26.0 ± 11.0%	24.6 ± 15.0%	11.1 ± 7.8%
1.25, 90	6.3 ± 8.8%	19.9 ± 10.1%	−23.1 ± 8.6%	32.2 ± 9.8%	−3.5 ± 9.6%
1.25, 180	−5.5 ± 8.2%	24.5 ± 6.0%	−3.7 ± 10.0%	62.2 ± 6.4%	−21.3 ± 11.0%
1.25, 270	11.1 ± 8.4%	38.4 ± 7.8%	−29.5 ± 7.3%	66.0 ± 8.6%	−3.1 ± 13.6%
2.5, 0	9.8 ± 6.8%	9.9 ± 4.8%	−6.0 ± 11.7%	29.6 ± 9.3%	10.9 ± 9.5%
2.5, 45	9.6 ± 9.3%	31.1 ± 5.1%	−22.2 ± 9.2%	5.2 ± 7.3%	−2.5 ± 10.7%
2.5, 90	−3.6 ± 9.4%	36.0 ± 6.0%	−28.5 ± 6.5%	31.9 ± 8.1%	−5.2 ± 9.1%
2.5, 135	13.9 ± 7.7%	24.0 ± 4.4%	−3.6 ± 8.6%	32.7 ± 9.9%	−7.1 ± 8.2%
2.5, 180	2.8 ± 11.1%	31.1 ± 6.9%	4.5 ± 7.6%	43.1 ± 12.7%	−7.6 ± 8.4%
2.5, 225	−5.3 ± 7.1%	14.7 ± 6.3%	21.5 ± 8.0%	65.7 ± 6.1%	−7.1 ± 7.8%
2.5, 270	1.9 ± 7.9%	11.6 ± 5.9%	−15.2 ± 9.2%	58.2 ± 9.2%	−15.1 ± 12.4%
2.5, 315	12.0 ± 8.2%	−1.2 ± 6.5%	−29.8 ± 7.8%	22.6 ± 8.4%	7.8 ± 9.0%
**Location**	**S06**	**S07**	**S08**	**S09**	**S10**
0, n/a	−11.4 ± 15.7%	27.2 ± 8.6%	6.0 ± 14.1%	60.8 ± 20.4%	−20.1 ± 11.5%
1.25, 0	−12.1 ± 11.1%	34.6 ± 8.1%	−11.6 ± 7.7%	−11.6 ± 16.8%	−6.9 ± 12.6%
1.25, 90	−10.2 ± 11.2%	8.9 ± 9.2%	−3.2 ± 8.1%	41.7 ± 16.8%	−27.9 ± 19.2%
1.25, 180	−52.1 ± 13.9%	14.1 ± 9.9%	−1.0 ± 10.7%	34.9 ± 10.5%	−8.9 ± 10.2%
1.25, 270	−37.2 ± 15.2%	21.0 ± 9.0%	−7.7 ± 8.7%	34.8 ± 16.8%	−8.4 ± 12.1%
2.5, 0	−22.6 ± 11.4%	6.5 ± 6.7%	−13.1 ± 9.1%	−8.6 ± 9.3%	23.1 ± 9.8%
2.5, 45	−13.2 ± 9.7%	22.4 ± 8.4%	7.6 ± 8.1%	29.5 ± 15.0%	−2.0 ± 7.0%
2.5, 90	8.8 ± 14.7%	14.5 ± 8.5%	−6.5 ± 7.9%	19.7 ± 13.4%	5.8 ± 10.1%
2.5, 135	−12.7 ± 9.2%	4.3 ± 8.1%	−8.4 ± 8.2%	22.4 ± 11.6%	30.3 ± 10.6%
2.5, 180	−40.6 ± 9.7%	21.3 ± 9.6%	3.1 ± 9.3%	35.7 ± 11.1%	7.0 ± 8.1%
2.5, 225	1.3 ± 18.4%	25.8 ± 6.8%	8.5 ± 9.3%	−10.6 ± 13.4%	−14.6 ± 9.8%
2.5, 270	−20.2 ± 10.4%	1.5 ± 7.0%	−7.9 ± 7.2%	46.4 ± 10.8%	−3.3 ± 11.1%
2.5, 315	−21.1 ± 14.9%	2.1 ± 9.3%	−7.3 ± 7.3	18.3 ± 10.1%	−31.1 ± 9.5%
**Location**	**S11**	**S12**	**S13**	**S14**	**S15**
0, n/a	29.5 ± 12.1%	−24.4 ± 16.6%	−39.0 ± 12.8%	27.4 ± 13.1%	1.4 ± 14.4%
1.25, 0	33.0 ± 12.0%	15.7 ± 9.7%	−14.3 ± 6.7%	1.0 ± 6.7%	14.7 ± 7.6%
1.25, 90	44.0 ± 14.6%	−14.7 ± 9.0%	−21.0 ± 9.2%	7.0 ± 8.0%	11.8 ± 10.7%
1.25, 180	14.9 ± 9.3%	−9.2 ± 8.8%	21.3 ± 15.6%	18.8 ± 8.0%	0.8 ± 10.5%
1.25, 270	37.9 ± 9.6%	10.5 ± 11.8%	4.0 ± 7.7%	7.9 ± 9.6%	−6.4 ± 8.7%
2.5, 0	−4.5 ± 24.5%	11.7 ± 10.0%	−3.9 ± 10.4%	−5.1 ± 7.5%	5.4 ± 8.5%
2.5, 45	2.4 ± 11.1%	−6.5 ± 8.6%	−17.5 ± 8.8%	3.6 ± 5.8%	6.9 ± 8.5%
2.5, 90	15.2 ± 9.6%	−7.6 ± 9.6%	−10.9 ± 6.6%	12.0 ± 7.0%	2.6 ± 7.7%
2.5, 135	27.1 ± 10.1%	−1.9 ± 10.4%	12.8 ± 9.7%	15.8 ± 7.4%	0.1 ± 7.5%
2.5, 180	34.8 ± 8.0%	8.8 ± 10.4%	16.7 ± 9.5%	15.7 ± 9.2%	−14.0 ± 8.9%
2.5, 225	3.9 ± 9.7%	3.0 ± 10.8%	21.8 ± 7.3%	28.1 ± 11.2%	1.8 ± 8.3%
2.5, 270	23.1 ± 7.7%	26.0 ± 9.1%	−6.2 ± 6.9%	13.4 ± 7.6%	−2.7 ± 6.9%
2.5, 315	11.2 ± 8.0%	19.4 ± 8.5%	−14.2 ± 7.7%	2.2 ± 9.5%	−0.8 ± 6.5%
**Location**	**S16**	**S17**	**S18**	**S19**	**S20**
0, n/a	2.1 ± 11.3%	20.5 ± 12.6%	13.4 ± 5.6%	60.8 ± 7.8%	16.0 ± 24.4%
1.25, 0	5.5 ± 9.2%	2.7 ± 8.6%	12.3 ± 6.4%	36.7 ± 7.5%	−3.7 ± 10.4
1.25, 90	−0.2 ± 6.9%	28.6 ± 10.2%	19.2 ± 6.8%	23.7 ± 7.8%	−32.3 ± 14.5%
1.25, 180	2.7 ± 10.1	48.1 ± 6.4%	2.1 ± 5.2%	21.6 ± 7.9%	−12.7 ± 11.5%
1.25, 270	−1.2 ± 8.7%	20.9 ± 6.9%	16.8 ± 5.0%	21.4 ± 8.2%	1.0 ± 10.3%
2.5, 0	−0.1 ± 6.0%	11.8 ± 6.5%	25.3 ± 5.4%	22.7 ± 7.0%	6.7 ± 8.5%
2.5, 45	8.0 ± 6.1%	12.3 ± 9.0%	1.1 ± 5.9%	23.5 ± 5.7%	8.8 ± 7.9%
2.5, 90	0.4 ± 7.0%	7.3 ± 10.6%	11.3 ± 5.6%	23.7 ± 5.9%	−32.9 ± 10.5%
2.5, 135	3.5 ± 6.9%	11.7 ± 8.2%	15.3 ± 5.6%	10.9 ± 9.4%	−5.0 ± 7.3%
2.5, 180	−14.9 ± 6.4%	37.9 ± 6.3%	8.2 ± 4.7%	27.8 ± 6.9%	−10.8 ± 6.7%
2.5, 225	5.3 ± 7.6%	19.3 ± 6.8%	20.9 ± 6.3%	21.9 ± 6.5%	−20.6 ± 7.9%
2.5, 270	−4.7 ± 7.5%	0.2 ± 7.8%	10.2 ± 5.4%	7.5 ± 7.8%	−1.8 ± 13.1%
2.5, 315	3.1 ± 7.3%	6.9 ± 5.7%	11.4 ± 4.7%	−6.1 ± 8.2%	−9.9 ± 8.0%
**Location**	**S21**	**S22**	**S23**	**S24**	**S25**
0, n/a	−11.3 ± 7.0%	25.8 ± 9.3%	−2.5 ± 12.8%	36.4 ± 12.7%	3.9 ± 14.6%
1.25, 0	22.5 ± 5.2%	7.5 ± 8.0%	31.3 ± 12.2%	22.2 ± 11.7%	1.6 ± 8.3%
1.25, 90	−9.1 ± 6.4%	−1.8 ± 9.9%	6.1 ± 7.8%	5.8 ± 10.1%	−0.4 ± 8.3%
1.25, 180	−3.5 ± 5.2%	19.3 ± 8.1%	−14.3 ± 7.7%	11.6 ± 10.7%	6.5 ± 7.0%
1.25, 270	9.3 ± 5.6%	10.3 ± 7.5%	−9.6 ± 8.4%	3.9 ± 10.3%	10.1 ± 10.6%
2.5, 0	13.7 ± 4.3%	27.2 ± 8.6%	28.5 ± 8.4%	22.7 ± 9.0%	9.2 ± 8.5%
2.5, 45	−5.0 ± 4.4%	8.0 ± 7.3%	5.3 ± 7.6%	−0.7 ± 9.2%	−10.2 ± 9.2%
2.5, 90	12.5 ± 4.3%	22.7 ± 7.6%	−3.0 ± 9.4%	−16.7 ± 7.2%	11.3 ± 7.2%
2.5, 135	−6.0 ± 4.9%	22.5 ± 7.1%	−9.2 ± 8.5%	3.8 ± 7.9%	−7.4 ± 7.9%
2.5, 180	6.4 ± 5.5%	11.5 ± 7.4%	−18.4 ± 9.0%	9.5 ± 7.8%	10.1 ± 6.3%
2.5, 225	9.1 ± 4.4%	10.3 ± 6.7%	9.9 ± 8.9%	36.0 ± 9.1%	8.2 ± 6.2%
2.5, 270	14.3 ± 4.2%	−12.5 ± 9.3%	17.8 ± 9.6%	16.3 ± 9.8%	12.5 ± 9.2%
2.5, 315	15.0 ± 4.8%	−8.6 ± 7.3%	3.5 ± 8.6%	13.6 ± 8.2%	9.0 ± 8.0%
**Location**	**S26**				
0, n/a	7.4 ± 13.2%				
1.25, 0	17.8 ± 11.7%				
1.25, 90	6.9 ± 10.5%				
1.25, 180	32.9 ± 8.6%				
1.25, 270	7.8 ± 9.3%				
2.5, 0	12.5 ± 8.5%				
2.5, 45	2.5 ± 8.9%				
2.5, 90	16.0 ± 9.8%				
2.5, 135	13.7± 9.6%				
2.5, 180	12.7 ± 8.2%				
2.5, 225	31.3 ± 9.3				
2.5, 270	6.8 ± 12.1%				
2.5, 315	−6.5 ± 9.2				

**Table A2:** This table contains the mean and standard deviation color dominance values corresponding to the individual heat maps in Figure 5. To compute standard deviation, we drew 10,000 bootstrap samples of green image and red image durations from each zone’s green and red data pool for each individual observer. The number of data points in the green and red samples were always matched to the observed number of green and red data points in each zone [16]. In the table below, eccentricity (ecc) is in degrees of visual angle. The direction (dir) refers to the position of the rival patches relative to fixation, with 0 directly to the left of fixation, and increasing numbers moving clockwise around a circle. In other words, 0 corresponds to West on a compass, 90 to North, etc. Positive numbers correspond to red predominance; negative numbers to green.

**Location (ecc, dir)**	**S01**	**S02**	**S03**	**S04**	**S05**
0, n/a	22.0 ± 11.2%	5.6 ± 7.8%	51.8 ± 8.5%	17.6 ± 13.0%	99.5 ± 0.1%
1.25, 0	12.5 ± 6.8%	15.2 ± 6.4%	33.4 ± 10.2%	−29.9 ± 10.9%	24.4 ± 6.6%
1.25, 90	18.0 ± 8.2%	−9.5 ± 9.9%	28.5 ± 8.5%	−11.8 ± 13.1%	31.5 ± 7.0%
1.25, 180	8.6 ± 8.2%	5.8 ± 6.7%	37.4 ± 7.7%	−1.8 ± 12.8%	29.5 ± 9.8%
1.25, 270	17.4 ± 7.7%	18.7 ± 9.3%	28.5 ± 7.3%	38.9 ± 15.4%	53.5 ± 7.0%
2.5, 0	18.6 ± 6.4%	−6.0 ± 4.8%	13.0 ± 11.4%	6.1 ± 10.7%	30.9 ± 8.2%
2.5, 45	10.5 ± 9.5%	6.4 ± 6.4%	13.3 ± 9.0%	−7.7 ± 7.3%	7.6 ± 10.7%
2.5, 90	16.3 ± 9.2%	−10.6 ± 7.8%	9.0 ± 6.8%	11.3 ± 9.0%	18.9 ± 8.2%
2.5, 135	16.4 ± 8.2%	5.0 ± 4.8%	24.4 ± 7.9%	34.9 ± 12.1%	25.0 ± 7.2%
2.5, 180	8.8 ± 11.2%	15.1 ± 8.9%	15.9 ± 7.2%	27.0 ± 11.3%	27.6 ± 6.7%
2.5, 225	20.5 ± 6.4%	19.8 ± 6.3%	21.0 ± 7.9%	21.0 ± 11.0%	20.3 ± 7.1%
2.5, 270	23.7 ± 7.0%	9.1 ± 5.8%	21.8 ± 8.4%	38.4 ± 11.5%	53.4 ± 6.8%
2.5, 315	21.3 ± 8.0%	6.8 ± 6.2%	34.8 ± 7.1%	2.2 ± 9.7%	29.0 ± 7.7%
**Location (ecc, dir)**	**S06**	**S07**	**S08**	**S09**	**S10**
0, n/a	−52.6 ± 11.1%	−27.0 ± 8.2%	−12.6 ± 14.0%	31.6 ± 28.8%	31.9 ± 11.5%
1.25, 0	7.1 ± 11.6%	−15.1 ± 9.6%	−17.1 ± 7.2%	7.8 ± 18.4%	28.0 ± 12.2%
1.25, 90	12.0 ± 10.5%	−10.8 ± 9.3%	−13.2 ± 7.7%	−45.4 ± 14.9%	41.5 ± 17.8%
1.25, 180	−19.0 ± 18.0%	−19.7 ± 8.8%	−24.3 ± 9.8%	11.3 ± 13.9%	1.5 ± 10.6%
1.25, 270	11.8 ± 21.6%	−19.8 ± 9.2%	4.1 ± 8.7%	41.5 ± 14.1%	9.4 ± 11.9%
2.5, 0	30.5 ± 10.1%	−13.8 ± 6.5%	−14.1 ± 9.7%	−1.8 ± 9.2%	13.7 ± 11.4%
2.5, 45	11.9 ± 10.4%	−0.9 ± 9.6%	−23.0 ± 7.8%	−36.0 ± 11.4%	18.5 ± 6.7%
2.5, 90	63.7 ± 8.8%	−18.8 ± 8.1%	−15.8 ± 7.8%	−7.4 ± 13.3%	−1.7 ± 9.6%
2.5, 135	17.4 ± 8.8%	−16.6 ± 7.6%	−24.2 ± 7.0%	−9.1 ± 12.3%	10.7 ± 12.3%
2.5, 180	3.8 ± 13.2%	−13.9 ± 10.2%	−22.6 ± 8.1%	2.9 ± 15.3%	−0.4 ± 8.2%
2.5, 225	40.9 ± 11.8%	0.8 ± 7.9%	−17.0 ± 9.1%	−7.0 ± 12.6%	15.9 ± 10.6%
2.5, 270	44.8 ± 8.8%	−2.9 ± 7.0%	−3.4 ± 7.3%	7.4 ± 15.5%	20.8 ± 10.6%
2.5, 315	14.2 ± 16.0%	−1.3 ± 9.2%	8.0 ± 7.2%	35.2 ± 8.6%	−10.8 ± 12.4%
**Location (ecc, dir)**	**S11**	**S12**	**S13**	**S14**	**S15**
0, n/a	43.1 ± 9.0%	−39.8 ± 11.8%	17.8 ± 16.7%	42.7 ± 11.3%	65.9 ± 6.7%
1.25, 0	28.5 ± 12.3%	−13.6 ± 9.9%	0.2 ± 7.0%	20.7 ± 6.0%	19.8 ± 7.4%
1.25, 90	38.8 ± 15.5%	−1.0 ± 9.5%	−0.5 ± 10.8%	21.9 ± 7.4%	33.1 ± 8.8%
1.25, 180	23.4 ± 8.7%	−13.7 ± 8.6%	15.4 ± 16.6%	17.1 ± 8.6%	45.5 ± 7.5%
1.25, 270	45.3 ± 7.7%	−11.0 ± 11.3%	18.1 ± 7.6%	36.0 ± 7.3%	37.8 ± 6.0%
2.5, 0	49.6 ± 15.7%	−1.4 ± 10.3%	10.7 ± 10.3%	7.6 ± 7.5%	26.5 ± 6.9%
2.5, 45	19.8 ± 10.0%	7.0 ± 8.6%	4.6 ± 10.1%	15.7 ± 5.5%	26.1 ± 7.4%
2.5, 90	3.9 ± 10.2%	−9.2 ± 9.5%	0.7 ± 7.1%	−5.6 ± 7.0%	19.9 ± 7.0%
2.5, 135	−0.4 ± 11.6%	−10.0 ± 10.3%	6.1 ± 10.3%	35.9 ± 6.7%	38.5 ± 5.7%
2.5, 180	30.0 ± 9.7%	−18.3 ± 9.5%	7.2 ± 10.7%	27.4 ± 7.8%	27.7 ± 8.5%
2.5, 225	19.3 ± 8.4%	12.2 ± 10.7%	22.4 ± 7.7%	45.2 ± 8.7%	36.2 ± 6.6%
2.5, 270	12.6 ± 7.2%	−12.6 ± 9.3%	−7.8 ± 7.0%	39.9 ± 5.3%	34.2 ± 5.2%
2.5, 315	18.7 ± 7.4%	−5.3 ± 9.0%	−18.0 ± 7.5%	26.9 ± 8.0%	34.5 ± 5.3%
**Location (ecc, dir)**	**S16**	**S17**	**S18**	**S19**	**S20**
0, n/a	51.0 ± 6.4%	−34.9 ± 10.5%	−16.6 ± 5.3%	48.9 ± 14.8%	77.1 ± 6.8%
1.25, 0	37.2 ± 6.3%	22.5 ± 8.3%	−1.3 ± 6.5%	14.0 ± 9.3%	29.7 ± 8.1%
1.25, 90	21.6 ± 6.4%	−12.9 ± 10.6%	9.0 ± 7.3%	36.2 ± 6.2%	32.0 ± 14.5%
1.25, 180	48.4 ± 5.8%	4.9 ± 8.9%	13.3 ± 5.1%	29.7 ± 7.6%	20.5 ± 10.9%
1.25, 270	40.1 ± 5.9%	−4.1 ± 7.5%	−10.2 ± 5.2%	32.2 ± 7.2%	34.7 ± 7.2%
2.5, 0	18.2 ± 5.5%	14.6 ± 6.3%	−13.5 ± 5.8%	9.3 ± 6.8%	11.3 ± 8.1%
2.5, 45	27.1 ± 5.1%	−11.9 ± 9.0%	−1.6 ± 5.9%	17.8 ± 6.1%	9.5 ± 8.0%
2.5, 90	14.9 ± 6.7%	−11.3 ± 10.5%	−8.5 ± 5.7%	13.5 ± 6.0%	16.9 ± 13.8%
2.5, 135	11.1 ± 6.6%	−4.2 ± 8.2%	−11.1 ± 5.9%	29.5 ± 8.8%	11.8 ± 7.0%
2.5, 180	22.2 ± 6.2%	25.0 ± 6.1%	−13.1 ± 4.6%	24.3 ± 7.1%	5.8 ± 6.7%
2.5, 225	25.3 ± 6.4%	13.9 ± 6.4%	8.4 ± 6.6%	2.8 ± 7.1%	4.0 ± 8.7%
2.5, 270	24.2 ± 6.1%	13.5 ± 7.1%	−11.1 ± 5.4%	45.0 ± 5.8%	15.7 ± 12.3%
2.5, 315	44.2 ± 4.9%	−0.3 ± 5.7%	−17.7 ± 4.4%	21.3 ± 7.7%	−0.4 ± 8.4%
**Location (ecc, dir)**	**S21**	**S22**	**S23**	**S24**	**S25**
0, n/a	−1.7 ± 7.2%	24.4 ± 10.0%	50.3 ± 9.2%	37.9 ± 10.9%	58.9 ± 8.1%
1.25, 0	7.0 ± 5.8%	17.2 ± 7.9%	24.6 ± 13.7%	52.0 ± 7.9%	31.1 ± 6.6%
1.25, 90	10.1 ± 6.1%	21.5 ± 9.4%	17.2 ± 7.7%	32.1 ± 10.1%	30.1 ± 6.7%
1.25, 180	−16.3 ± 4.9%	26.1 ± 7.8%	30.5 ± 7.6%	47.8 ± 7.1%	19.9 ± 6.3%
1.25, 270	−13.8 ± 5.4%	25.9 ± 7.6%	23.7 ± 8.2%	33.7 ± 7.9%	33.4 ± 8.2%
2.5, 0	9.8 ± 4.5%	17.5 ± 9.3%	24.0 ± 9.7%	20.3 ± 9.3%	29.2 ± 7.0%
2.5, 45	−8.0 ± 4.3%	21.4 ± 7.4%	6.2 ± 7.3%	37.8 ± 7.1%	41.6 ± 6.9%
2.5, 90	6.9 ± 4.4%	22.3 ± 7.7%	−0.6 ± 9.1%	31.1 ± 7.2%	27.8 ± 6.6%
2.5, 135	−3.2 ± 4.9%	17.9 ± 7.0%	12.4 ± 8.5%	30.3 ± 6.7%	27.1 ± 6.4%
2.5, 180	−4.5 ± 5.5%	25.2 ± 6.4%	18.1 ± 9.4%	32.4 ± 6.0%	12.9 ± 6.2
2.5, 225	−0.2 ± 4.5%	24.7 ± 6.8%	26.8 ± 8.4%	27.7 ± 10.0%	27.5 ± 4.9%
2.5, 270	−2.8 ± 4.7%	15.2 ± 10.2%	36.6 ± 7.7%	19.8 ± 10.3%	35.1 ± 7.0%
2.5, 315	−1.5 ± 5.2%	9.1 ± 7.4%	15.9 ± 8.2%	32.6 ± 7.1%	20.0 ± 7.8%
**Location (ecc, dir)**	**S26**	**S27**			
0, n/a	44.2 ± 8.7%	51.1 ± 15.3			
1.25, 0	27.6 ± 10.2%	15.9 ± 8.0%			
1.25, 90	21.0 ± 8.8%	14.9 ± 8.2%			
1.25, 180	23.3 ± 9.7%	12.2 ± 8.5%			
1.25, 270	26.9 ± 7.4%	12.3 ± 8.0%			
2.5, 0	−5.1 ± 9.1%	8.5 ± 7.6%			
2.5, 45	15.0 ± 8.4%	−8.7 ± 7.6%			
2.5, 90	17.8 ± 9.6%	−12.4 ± 7.5%			
2.5, 135	7.3 ± 10.7%	−26.1 ± 6.7%			
2.5, 180	11.6 ± 8.2%	2.0 ± 9.6%			
2.5, 225	16.8 ± 10.7%	6.2 ± 7.0%			
2.5, 270	34.3 ± 8.9%	3.8 ± 6.4%			
2.5, 315	4.4 ± 9.3%	7.4 ± 7.1%			
